# Genome-wide identification of the *EXO70* genes to elucidate their potential roles for intraspecific cross-incompatibility in sweet potato (*Ipomoea batatas* L.)

**DOI:** 10.3389/fpls.2026.1756265

**Published:** 2026-02-26

**Authors:** Xiaoyu Zhang, Ruidong Zeng, Bingzhi Jiang, Hongda Zou, Xiangbo Zhang, Rong Zhang, Chaocheng Tang, Zhufang Yao, Zhongxia Luo, Lifei Huang, Faqiang Feng, Zhangying Wang, Yiling Yang

**Affiliations:** 1 Guangdong Provincial Key Laboratory of Crop genetic Improvement, Crops Research Institute, Guangdong Academy of Agricultural Sciences, Guangzhou, China; 2 College of Agriculture, South China Agricultural University, Guangzhou, China

**Keywords:** breeding, EXO70 gene, genome-wide, intraspecific cross-incompatibility, sweet potato

## Abstract

Crossbreeding is the primary approach for sweet potato improvement, however, frequent cross-incompatibility during intraspecific hybridization remains a major bottleneck in breeding programs. EXO70 proteins, which regulate vesicle secretion during pollen germination, have been reported to play important roles in self-incompatibility in *Brassicaceae*. To investigate the potential involvement of *EXO70* genes in intraspecific cross-incompatibility in sweet potato, members of the *EXO70* gene family were systematically identified from the sweet potato genome and analyzed for their molecular characteristics as well as expression patterns across different tissues. A total of 35 *EXO70* genes *(IbEXO70)* were identified in sweet potato (*Ipomoea batatas*). Phylogenetic analysis classified these genes into three branches and nine subgroups, showing similar gene number and subgroup distributions to those in the diploid wild relatives of *Ipomoea trifida* and *Ipomoea triloba*, although differences were observed in chromosomal distribution and conserved protein motif composition. Gene structure analysis revealed that members of the *IbEXO70A* subgroup contained a higher number of exons and introns. Tissue specific expression profiling indicated that nine *IbEXO70* genes were significantly upregulated in compatibly pollinated stigmas compared with incompatible or unpollinated stigmas. Among these, *IbEXO70-26*, belonging to the *EXO70H* subgroup, was identified as a strong candidate regulator of cross-incompatibility due to its highest and stigmas and pollen specific expression, particularly under compatible pollination conditions. These results were further supported by transcriptomic comparisons between compatible and incompatible samples, and subcellular localization analysis showed that *IbEXO70–26* protein was localized to the nucleus. This study provides a comprehensive characterization of the *EXO70* gene family in sweet potato and lays a solid foundation for future functional studies of compatibility factors involved in pollen-stigma interactions.

## 1 Introduction

Sweet potato (*Ipomoea batatas* L.) Lam.) is a hexaploid crop valued for its high yield, wide adaptability, and rich nutritional composition. As one of the world’s seven major food crops, it plays an important role as a source of human food, animal feed, and industrial raw materials (Zhang et al., 2003; Drapal et al., 2019; Fan et al., 2021; Weng et al., 2023). At present, crossbreeding remains the primary strategy for sweet potato improvement. However, intraspecific cross-incompatibility (ICI) frequently occurs during hybridization, severely restricting parental combinations and posing a major obstacle to the development of sweet potato breeding programs (Yang et al., 2022). Based on the ICI, the sweet potato has been divided into 16 incompatibility groups (A to O and X), with varieties within the same group exhibiting cross-incompatibility, while varieties from different groups are compatible (Shen, 1984). Studies have shown that the phenotype of ICI is characterized by the inability of the pollen to germinate on the stigma in incompatible combinations. This phenomenon is attributed to the accumulation of callose on the stigma and resembling the phenotype of sporophytic self-incompatibility (SSI) (Lu and Tang, 1992; Ketong Wang, 1992).

SSI in *Brassicaceae* has been widely studied and is regulated by SCR (S-locus cysteine-rich protein) and SRK (S-locus receptor kinase) (Fujii et al., 2016). In this system, recognition of an incompatible pollen by the stigma triggers an interaction between the SCR ligand and the SRK receptor, leading to SRK phosphorylation. The activation of SRK subsequently recruits the downstream MDPK (M-locus protein kinase), which then phosphorylates ARC1 (Arm repeat containing 1) (Cabrillac et al., 2001; Takayama et al., 2001; Iwano et al., 2003; Murase et al., 2004). As an E3 ubiquitin ligase, ARC1 mediates the ubiquitination and hydrolysis of EXO70, a key pollen recognition factor thereby disrupting the transport of secretory vesicles, impairing pollen hydration and ultimately inhibiting pollen tube germination (He et al., 2007; Samuel et al., 2009; Safavian and Goring, 2013; Safavian et al., 2014).

While previous research on *Ipomoea trifida*, a diploid relative of sweet potato, have revealed that SRK is not the key regulator for incompatibility, as evidenced by homologous cloning and RFLP verification (Kowyama et al., 1995; Kowyama et al., 1996; Kowyama et al., 2000). To identify the genes involved in regulating incompatibility, Rahman et al. employed map-based cloning and genetic recombination approaches to locate S-locus region and screened three stigma specific genes *(SE1*, *SE2*, and *SEA3)* and one anther specific gene *(AB2)* that are related to incompatibility of *Ipomoea trifida* (Tomita et al., 2004; Rahman et al., 2007). However, functional validation of these genes in context of incompatibility has not yet been reported and the underlying molecular mechanisms and regulatory networks of sweet potato ICI remain poorly understood.

To further explore the genetic basis of ICI, we focused on the phenotype of SSI, in which secretion of hydrated materials on the stigma surface plays a crucial role in pollen germination (Qu et al., 2015). Exocytosis is an important pathway mediating the secretion of these hydrated materials. *EXO70*, a critical subunit of the exocyst complex, plays a pivotal role in vesicle trafficking and exocytosis and is evolutionarily conserved across eukaryotes, including yeast, mammals, and plants (Synek et al., 2006; Chong et al., 2010; Ma et al., 2016; Zhu et al., 2019). In land plants, the *EXO70* gene family has undergone extensive expansion, resulting in numerous paralogues that are classified into three major clades, EXO70.1, EXO70.2, and EXO70.3 and further subdivided into EXO70A-I subgroups, which participate in diverse processes of plant growth and development (Fendrych et al., 2010; Zarsky et al., 2020). EXO70A1 has been identified as a key determinant of compatibility in the *Brassicaceae* SSI system. Loss of *EXO70A1* causes the stigma to reject compatible pollen, whereas its overexpression can overcome self-pollen rejection (Samuel et al., 2009; Cvrckova et al., 2012). In *Arabidopsis thaliana*, the disruption of *AtEXO70A2* reduces pollen germination efficiency and impairs pollen tube growth (Marković et al., 2020). Similarly, *EXO70C2* functions as a major regulator of apical growth in *A. thaliana* pollen tubes by controlling vesicle secretion rates, and loss of *EXO70C2* results in abnormal pollen tube growth characterized by irregular elongation, insufficient cell wall deposition, and intermittent growth arrest (Synek et al., 2017).

Despite these advances, research on *EXO70* genes in sweet potato remains limited. In our preliminary study, transcriptome analysis of cross-compatible and incompatible samples revealed that multiple *EXO70* genes exhibit differential expression response to ICI suggesting a potential regulatory role for *EXO70* family members in sweet potato cross-incompatibility (Yang et al., 2022). Therefore, the objectives of the present study were to: (1) comprehensively identify *EXO70* family genes in the sweet potato genome, and (2) systematically characterize their structural features and expression patterns to identify candidate genes potentially involved in ICI regulation. The findings of this study will provide a foundation for future functional studies of *EXO70* genes and their roles in pollen-stigma interactions in sweet potato.

## 2 Material and methods

### 2.1 Plant materials

In this study, three sweet potato cultivars; ‘Guangshu 79’ (GS79), ‘Guangshu 146’ (GS149) and ‘Shangshu 19’ (SS19) were grown in the National Sweet Potato Germplasm Nursery (Guangzhou), China. ‘GS 79’ and ‘GS 146’ were developed by Crops Research Institute, Guangdong Academy of Agricultural Sciences, whereas SS 19 was bred by the Shangqiu Academy of Agricultural and Forestry Sciences. Among these cultivars, GS 146 (Maternal Parent) and GS 79 (Pollen parent) constituted a cross-compatible combination, while GS 146 (Maternal Parent) and SS 19 (Pollen parent) represented a cross-incompatible combination.

### 2.2 Identification and phylogenetic tree construction of *EXO70* family members

The coding sequences (CDS), protein sequences, whole genome sequences and GFF3 annotation files of *Ipomoea batatas* were obtained from the ‘Taizhong 6’ genome database (https://sweetpotao.com/download_genome.html) (Yang et al., 2017). The hidden Markov model (HMM) profile of EXO70 (PF03081) was downloaded from the Pfam database (http://pfam-legacy.xfam.org/) and used to identify candidate *EXO70* genes in the sweet potato genome using HMMER v3.3.3 software (Mistry et al., 2021). Redundant sequences were removed, and candidate genes were further validated using the CDD-research (https://www.ncbi.nlm.nih.gov/Structure/bwrpsb/bwrpsb.cgi) to confirm the presence of the conserved EXO70 domain, resulting in the final identification of *IbEXO70* family members. The CDS, protein sequences, whole genome sequences and GFF3 annotation files of the diploid *Ipomoea trifida* and *Ipomoea triloba* were downloaded from Sweet Potato Genomics Resource (http://sweetpotato.uga.edu/gt4sp_download.shtml) (Wu et al., 2018; Li et al., 2022; Du et al., 2023). *EXO70* gene family members in diploid genome were identified using the same pipeline. The EXO70 protein sequences of *Arabidopsis thaliana* were obtained from TAIR (https://www.*Arabidopsis*.org/) while those of *Oryza sativa* and *Brassica oleracea* were retrieved from Phytozome13 (https://phytozome-next.jgi.doe.gov/).

Multiple sequence alignment of EXO70 proteins from *I. batatas*, *I. trifida, I. triloba*, *A.thaliana* and *O. sativa* was performed using the Clustalw algorithm implemented in MEGA v6.0 (Thompson et al., 2002; Larkin et al., 2007; Tamura et al., 2013). Phylogenetic trees were constructed using the neighbor-joining (NJ) method with the Poisson correction model and branch support was evaluated with 1000 bootstrap replicates (Yang et al., 2017). The phylogenetic trees were visualized and optimized using ChiPlot (https://www.chiplot.online/).

### 2.3 Bioinformatics analysis of *EXO70* gene

The physicochemical properties of the EXO70 proteins from *I. batatas*, *I. trifida*, and *I. triloba*, including molecular weight and isoelectric point, were analyzed using ExPASy ProtParam (https://web.expasy.org/protparam/) (Wilkins et al., 1999). The subcellular localization of EXO70 proteins was predicted using WoLF PSORT (https://wolfpsort.hgc.jp/). Chromosomal distribution, gene structure (exon-intron organization), and visualization were performed using TBtools (Chen et al., 2020). Conserved protein motifs were identified using the MEME online suite and subsequently visualized with TBtools. The 2000 bp upstream promoter sequences of *IbEXO70* genes were extracted using TBtools, and cis-acting regulatory elements were predicted using PlantCARE (https://bioinformatics.psb.ugent.be/webtools/plantcare/html/) (Lescot et al., 2002; Chen et al., 2020). The distribution of *cis*-acting elements was visualized in TBtools.

To investigate the gene duplication events, MCScanX was used to identify tandem and segmental duplication events among *EXO70* genes in *I. batatas*, *I. trifida*, *I. triloba*, *A. thaliana*, and *O. sativa* (Wang et al., 2012). Syntenic relationships and collinearity of EXO70 genes across these species were visualized using TBtools.

### 2.4 Expression pattern analysis

To investigate the expression profiles of *IbEXO70* genes in different tissues, a total of 16 tissues types were collected from the grafted GS 146 plants, including petal, budding flower period, buds period, receptacle, stigma, pollen, the compatible pollinated stigma (GS 146 × GS 79), incompatible pollinated stigma (GS 146 × SS 19), as well as the functional leaves, newly expanded leaves, petioles and stems from both flowering and non-flowering plants.

Total RNA was extracted using the RNAprep Pure Polysaccharide Polyphenol Plant Total RNA Kit (TIANGEN, Beijing, China), and cDNA was synthesized with the HiScriptIII 1st Strand cDNA Synthesis Kit (TIANGEN, Beijing, China). Gene-specific primers were designed (**Supplementary Table S1**), and quantitative real-time PCR (qRT-PCR) was performed on a Bio-Rad fluorescence quantitative PCR (Bio-Rad CFX96,Bio-Rad Laboratories, Inc) system. Each sample was analyzed with three biological replicates, and the relative expression levels were calculated using the 2^-ΔΔCt^ method (Livak and Schmittgen, 2001).

### 2.5 Subcellular localization of *IbEXO70-26*

The full length coding sequence of *IbEXO70–26* was amplified and cloned into pCAMBIA1300-GFP vector. The recombinant construct was then introduced into *Agrobacterium tumefaciens* strain GV3101 and subsequently infiltrated into fully expanded leaves of healthy *Nicotiana benthamiana* plants. After infiltration, plants were incubated under low light conditions for 2 days. Infiltrated leaf sections (approximately 1 cm²) were then treated with 0.8 M mannitol for 1–2 h before microscopic observation. GFP fluorescence signals were examined using a Zeiss LSM880 laser scanning confocal microscope (Jena, Germany). Leaves infiltrated with the empty pCAMBIA1300-GFP vector were used as controls. Three independent biological replicates were performed.

### 2.6 Statistical analysis

All date were expressed as mean values calculated using WPS Office Excel. Statistical analyses were conducted using SPSS v22.0 software. One-way Analysis of Variance (ANOVA) was performed, and mean comparisons were conducted using the least significant difference (LSD) test at the *P* < 0.05 level. Differences among treatments were indicated by different lowercase letters, with letters from a to z representing descending significance level.

## 3 Results

### 3.1 Identification, chromosome mapping and characterization of *EXO70* family genes in *Ipomoea* species

**Figure 1:**
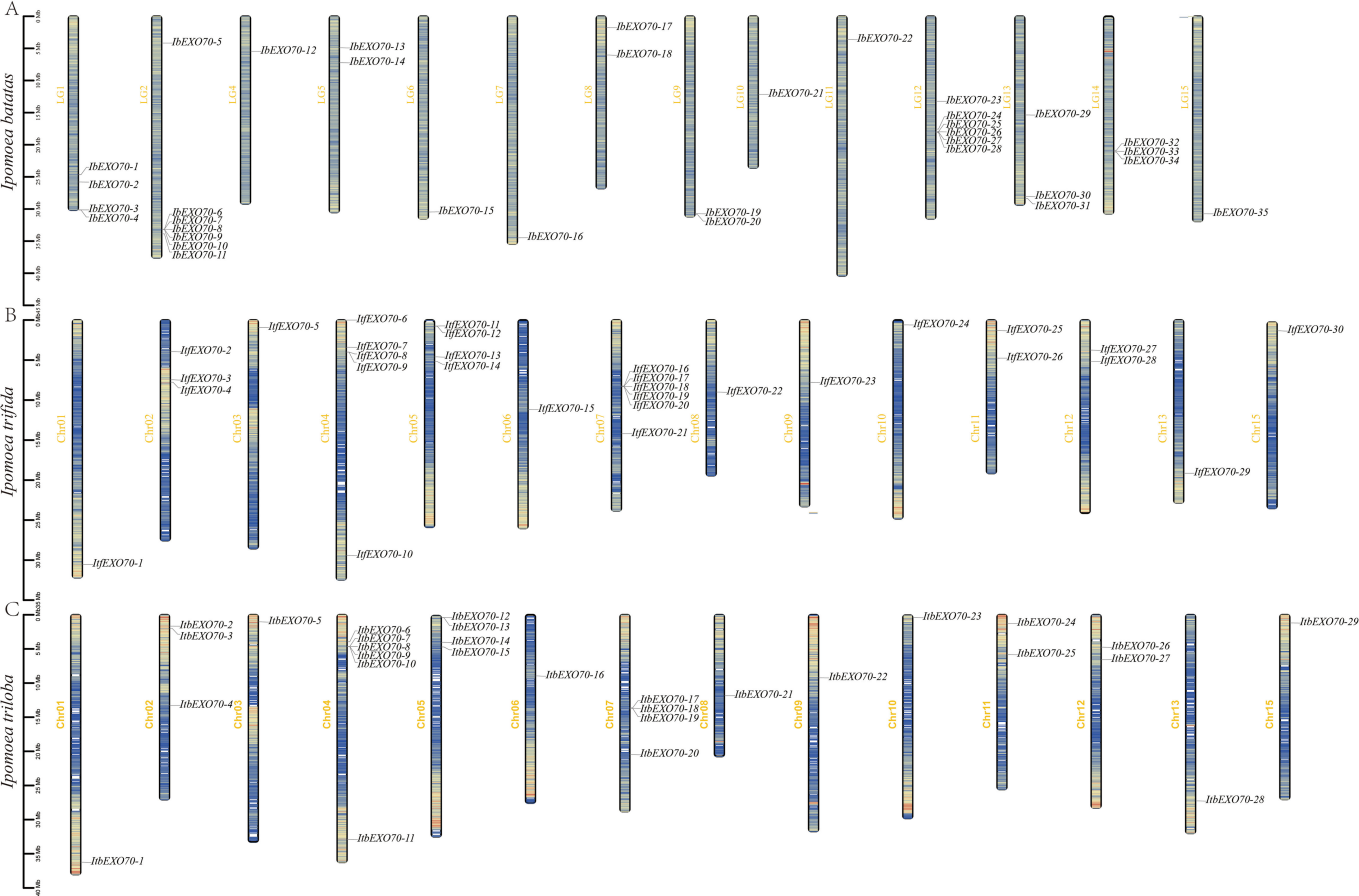
Chromosomal distribution of *EXO70* gene family members in three *Ipomoea* species. **(A)** Chromosome distribution map of *EXO70* in *I*. *batatas*. **(B)** Chromosome distribution map of *EXO70* in *I. trifida.***(C)** Chromosome distribution map of *EXO70* in *I*. *triloba*. The scale bar on the left represents the length of the chromosome.

A total of 94 *EXO70* genes were identified across three *Ipomoea* species, including 35 genes in the hexaploid *Ipomoea batatas*, and 30 and 29 genes in the diploid relatives *Ipomoea trifida* and *Ipomoea triloba*, respectively. The comparable numbers of *EXO70* genes among cultivated and wild sweet potato species indicate a high degree of conservation of the *EXO70* gene family in *Ipomoea*, with no extensive gene expansion following polyploidization. Chromosomal distributions indicated that *EXO70* genes were unevenly distributed across chromosomes (**Figure 1**). Interestingly, the distribution patterns of *ItfEXO70* and *ItbEXO70* genes were highly similar, with identical gene numbers and largely conserved chromosomal positions. Minor differences were observed, two additional *ItfEXO70* genes on chromosome 7, and the absence of one *ItfEXO70* gene on chromosome 4 (**Figures 1B, C**). In both diploid species, *EXO70* genes were mainly concentrated on chromosomes 2, 4, 5, and 7, with each chromosome harboring 3 to 6 genes.

In contrast, the *IbEXO70* genes in hexaploid sweet potato were distributed across all 15 chromosomes except chromosome 3 and were predominantly located on chromosomes 1, 2, 12, 13, and 14. These chromosomes contained 4, 7, 6, 3 and 3 *IbEXO70* genes, accounting for 11.42%, 20%, 17.14%, 8.57%, and 8.57% respectively (**Figure 1A**). The distinct chromosomal distribution patterns observed between hexaploid, and diploid *Ipomoea* species suggest potential divergence in gene regulation or functional specialization. Based on their chromosomal positions, *EXO70* genes were subsequently named *IbEXO70–1* to *35*, *ItfEXO70–1* to *30* and *ItbEXO70–1* to *29*.

Analysis of physicochemical properties of IbEXO70 proteins showed substantial variation in protein length and molecular characteristics (**Table 1****).***IbEXO70–1* was the shortest protein (406 amino acids), whereas *IbEXO70*-*17* (1163 amino acids) was the longest protein. The remaining members ranged between 438 and 933 amino acids. The predicted molecular weights ranged from 45.32 to 128.65 kDa, and the theoretical isoelectric points (pI) varied from 4.74 to 9.16. Subcellular localization prediction revealed that most IbEXO70 proteins were localized to the chloroplasts and the cytoplasm, except for IbEXO70–14 and IbEXO70-32, which were localized to the nucleus.

**Table 1:** List of detailed physicochemical properties of IbEXO70 proteins.

Gene	Sequence ID	Exon number	Number of amino acid	Molecular weight (kDa)	Theoretical pI	Subcellular location
*IbEXO70-1*	g3445.t1	8	406	45.32	7.7	Cytoplasm
*IbEXO70-2*	g3590.t1	2	623	69.77	6.21	Chloroplast
*IbEXO70-3*	g4175.t1	1	583	66.44	6.14	Chloroplast
*IbEXO70-4*	g4180.t1	1	658	74.00	4.98	Chloroplast
*IbEXO70-5*	g4806.t1	2	590	65.69	5.26	Chloroplast
*IbEXO70-6*	g8714.t1	1	568	65.18	6.15	Chloroplast
*IbEXO70-7*	g8715.t1	2	557	63.56	8.06	Chloroplast
*IbEXO70-8*	g8716.t1	1	559	63.81	8.16	Chloroplast
*IbEXO70-9*	g8717.t1	1	546	62.45	6.8	Chloroplast
*IbEXO70-10*	g8719.t1	3	713	80.46	8.8	Cytoplasm
*IbEXO70-11*	g8815.t1	3	551	62.45	5.94	Chloroplast
*IbEXO70-12*	g13597.t1	1	645	72.84	5.67	Chloroplast
*IbEXO70-13*	g17352.t1	5	753	85.48	4.85	Chloroplast
*IbEXO70-14*	g17674.t1	3	739	83.46	4.78	Nucleus
*IbEXO70-15*	g25071.t1	13	933	106.27	8.93	Chloroplast
*IbEXO70-16*	g30046.t1	1	658	75.25	4.74	Chloroplast
*IbEXO70-17*	g30560.t1	8	1163	128.65	6.75	Chloroplast
*IbEXO70-18*	g31290.t1	2	584	66.54	5.37	Chloroplast
*IbEXO70-19*	g38067.t1	3	620	70.16	7.97	Cytoplasm
*IbEXO70-20*	g38086.t1	5	668	75.49	7.58	Cytoplasm
*IbEXO70-21*	g39836.t1	1	674	76.52	5.67	Chloroplast
*IbEXO70-22*	g41951.t1	2	714	81.94	5.31	Cytoplasm
*IbEXO70-23*	g48659.t1	2	593	67.85	5.5	Chloroplast
*IbEXO70-24*	g49242.t1	1	480	53.50	5.51	Chloroplast
*IbEXO70-25*	g49297.t1	2	443	49.60	5.57	Chloroplast
*IbEXO70-26*	g49301.t1	1	477	53.27	6.07	Chloroplast
*IbEXO70-27*	g49304.t1	1	480	53.52	5.25	Chloroplast
*IbEXO70-28*	g49316.t1	2	438	48.49	6.05	Chloroplast
*IbEXO70-29*	g53255.t1	1	638	73.63	5.57	Chloroplast
*IbEXO70-30*	g55109.t1	3	605	68.49	5.37	Chloroplast
*IbEXO70-31*	g55167.t1	1	620	70.52	5.54	Chloroplast
*IbEXO70-32*	g58245.t1	12	722	81.97	9.16	Nucleus
*IbEXO70-33*	g58259.t1	11	612	69.42	8.52	Chloroplast
*IbEXO70-34*	g58277.t1	11	618	70.18	8.8	Chloroplast
*IbEXO70-35*	g64110.t1	2	622	70.82	6.06	Chloroplast

For diploid species, the amino acid lengths of ItfEXO70 proteins ranged from 449 to 808 (aa), with molecular weights of 50.11-92.42 kDa, and pI values of 4.61-9.89. The amino acid length of ItbEXO70 proteins ranged from 478 to 786 (aa), with molecular weights between 53.37 to 88.63 kDa, and the pI values ranging from 4.55 to 8.97. The results of subcellular localization prediction showed that both ItfEXO70 and ItbEXO70 proteins were predominantly localized to chloroplasts and the nucleus (**Supplementary Tables S2**, **S3**).

### 3.2 Phylogenetic analysis of *EXO70* genes in different species

**Figure 2:**
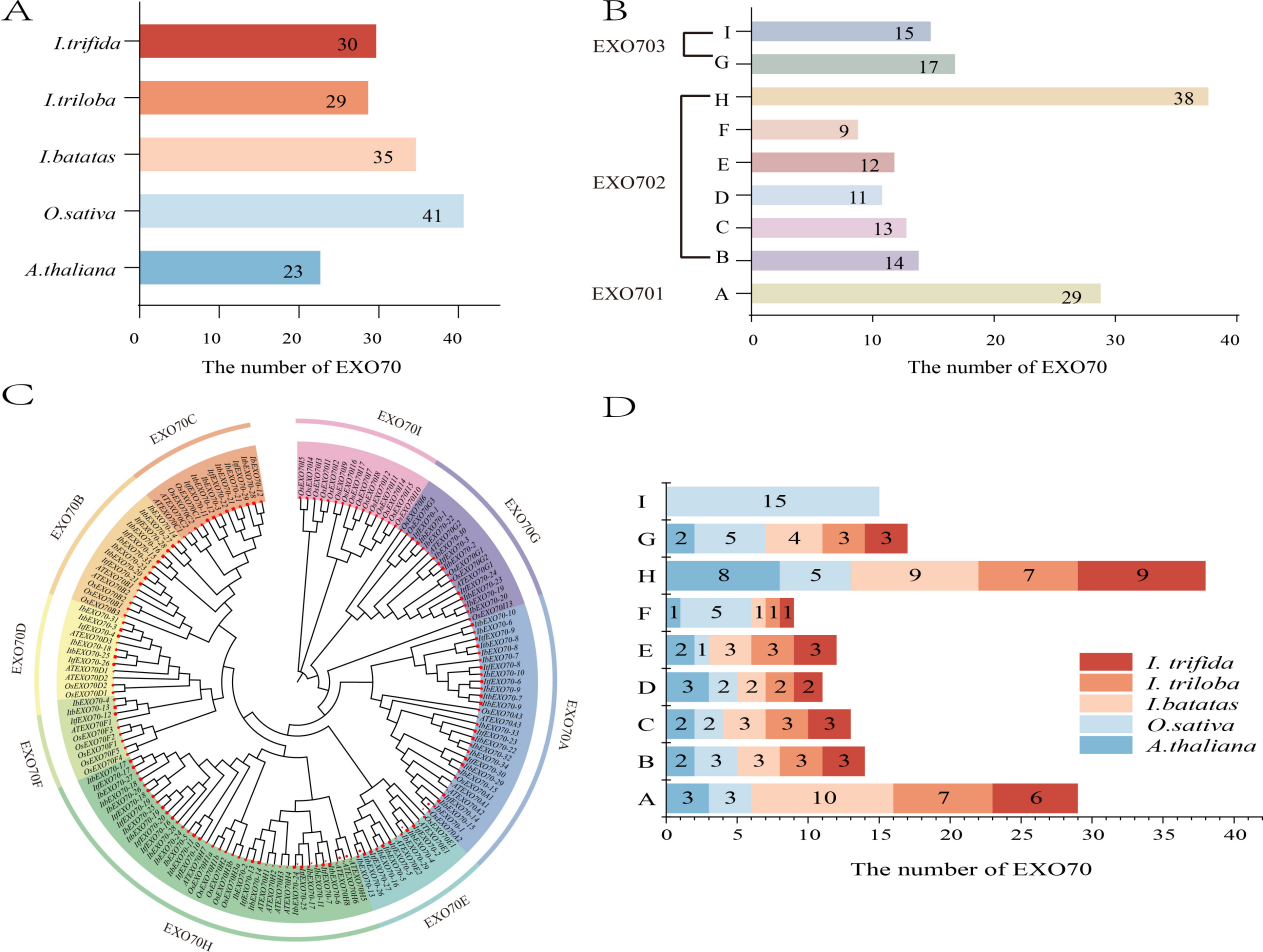
Phylogenetic analysis of the EXO70 gene family in different species. **(A)** The total number of *EXO70* genes in five species (*I. batatas*, *I. triloba*, *I. trifida*, *O. sativa*, and *A. thaliana)* The bar graph illustrates the variation in the number of EXO70 genes across these species, providing a comparative view of gene family size. **(B)** The distribution of *EXO70* genes in the subgroups from EXO70A to EXO70I. The bar graph shows the categorization of *EXO70* genes into subgroups, highlighting the relative proportions of each subgroup within the gene families of the five species. **(C)** The phylogenetic analysis of the *EXO70* gene family in *I. batatas*, *I. triloba*, *I. trifida*, *O. sativa*, and *A. thaliana* respectively. The phylogenetic tree, constructed based on sequence similarity, provides insights into the evolutionary relationships and classification of EXO70 genes in these species. **(D)** Number of *EXO70s* in each subgroup of *I. batatas*, *I. triloba*, *I. trifida*, *O. sativa*, and *A. thaliana* respectively. The bar graph shows the distribution of EXO70 genes across different subgroups in each species, highlighting differences in gene family composition. The numbers in panels **(A–C)** represent the number of EXO70.

To elucidate the evolutionary relationships of *EXO70* genes, a phylogenetic tree was constructed using *EXO70* protein sequences from *I. batatas* (35), *I. trifida* (30), *I. triloba* (29), *A. thaliana* (23) and *O. sativa* (41) (**Figure 2A**). The phylogenetic analysis classified all *EXO70* genes into three major clades, designated EXO70.1, EXO70.2, and EXO70.3, which were further subdivided into nine subgroups (EXO70A-EXO70I) (**Figure 2B**).

The EXO70.1 clade consisted solely of *EXO70A* subgroup (including 29 genes, accounting for 18.35% of the total EXO70 genes analyzed). The EXO70.2 clade contained six subgroups (EXO70B-EXO70F and EXO70H) containing 97 genes (61.40%), whereas the EXO70.3 clade included the remaining two subgroups, EXO70G and EXO70I, with a total of 32 genes (20.25%) (**Figure 2C**). Consistent with previous reports, the EXO70H subgroup showed evidence of rapid expansion in dicotyledonous plants (Sekereš et al., 2017) and contained the largest number of *EXO70* genes, followed by EXO70A, while EXO70F contained the fewest members.

The distribution of *EXO70* genes among subgroups was highly conserved across dicotyledonous species, particularly within *Ipomoea*. In the EXO70B-F subgroups gene numbers were nearly Identical among *Ipomoea* species (**Figure 2D**). Specifically, the EXO70B and EXO70F each contained one gene, EXO70D and EXO70E contained three genes, and EXO70C contained five genes. This conserved pattern indicates strong evolutionary stability of the *EXO70* gene family in *Ipomoea*. In contrast the monocotyledonous species *O. sativa* harbored a higher number of *EXO70* genes in the EXO70I subgroup. In previous studies, the EXO70I subgroup was commonly found in monocotyledonous plants (Chong et al., 2010). In this study, the absence of the EXO70I subfamily in dicotyledons indicates that EXO70 genes indeed exhibit differences between dicotyledons and monocotyledons.

### 3.3 Structural analysis of *EXO70* genes in *Ipomoea* species

**Figure 3:**
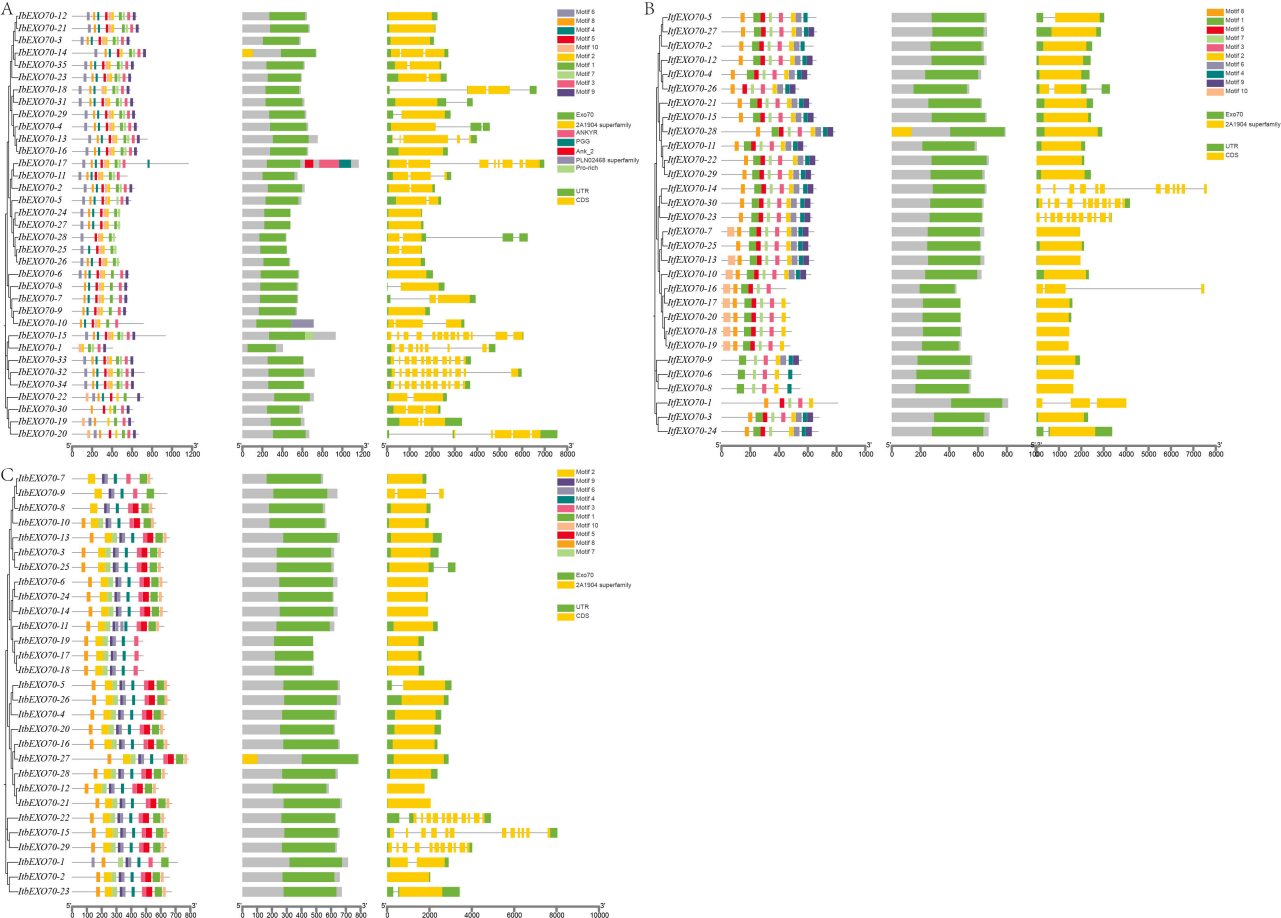
Visual representation of identified motifs, domains and exon/intron of the EXO70 gene family in Ipomoea species. **(A)** Motifs, domains and exon/intron identification of the *EXO70* gene family in ***I*.***batatas*. **(B)** Motifs, domains and exon/intron identification of the *EXO70* gene family in *I*. *trifida*. **(C)** Motifs, domains and exon/intron identification of the *EXO70* gene family in *I. triloba*. The figure illustrates the conserved motifs and domains across the *EXO70* genes in *I*. *batatas*, *I*. *trifida* and *I*. *triloba* as well as the exon/intron structure for each gene.

Based on the evolutionary relationships of *EXO70* genes in *Ipomoea* species, the conserved protein motifs and gene structures of *EXO70* family members were systematically analyzed (**Figure 3**). Motif analysis identified a total of ten conserved motifs among *EXO70* proteins. These motifs are widely distributed across most EXO70 members of the hexaploid species *I. batatas*, and diploid species *I. trifida* and *I. triloba*. Members of the *EXO70* gene family in sweet potato species all contain the conserved EXO70 domain. Analysis of exon/intron organization revealed substantial structural diversity among *EXO70* genes. Genes with relatively high numbers of exons and introns were predominantly clustered within the EXO70A subgroup. For instance, *IbEXO70–15* contained 13 exons, while *ItfEXO70–14* and *ItbEXO70–15* each harbored 12 exons. In contrast, *EXO70* genes belonging to the other subgroups generally contained possessed only 1 to 3 exons. Notable structural variations were also observed among genes within the same subgroup. For example, within the EXO70H subgroup, *IbEXO70–17* contained 8 exons, whereas *ItfEXO70–10* and *ItbEXO70–11* contained only 1 exon. Similarly, in the EXO70E subgroup, *IbEXO70–13* contained 5 exons, while all other members of this subgroup possessed only one exon. These results indicate that *EXO70* genes within the same subgroup exhibit considerable divergence in gene structures, which may reflect functional diversification or linear specific evolutionary adaptation.

### 3.4 Analysis of *cis*-acting elements of *I. batatas EXO70* genes

**Figure 4:**
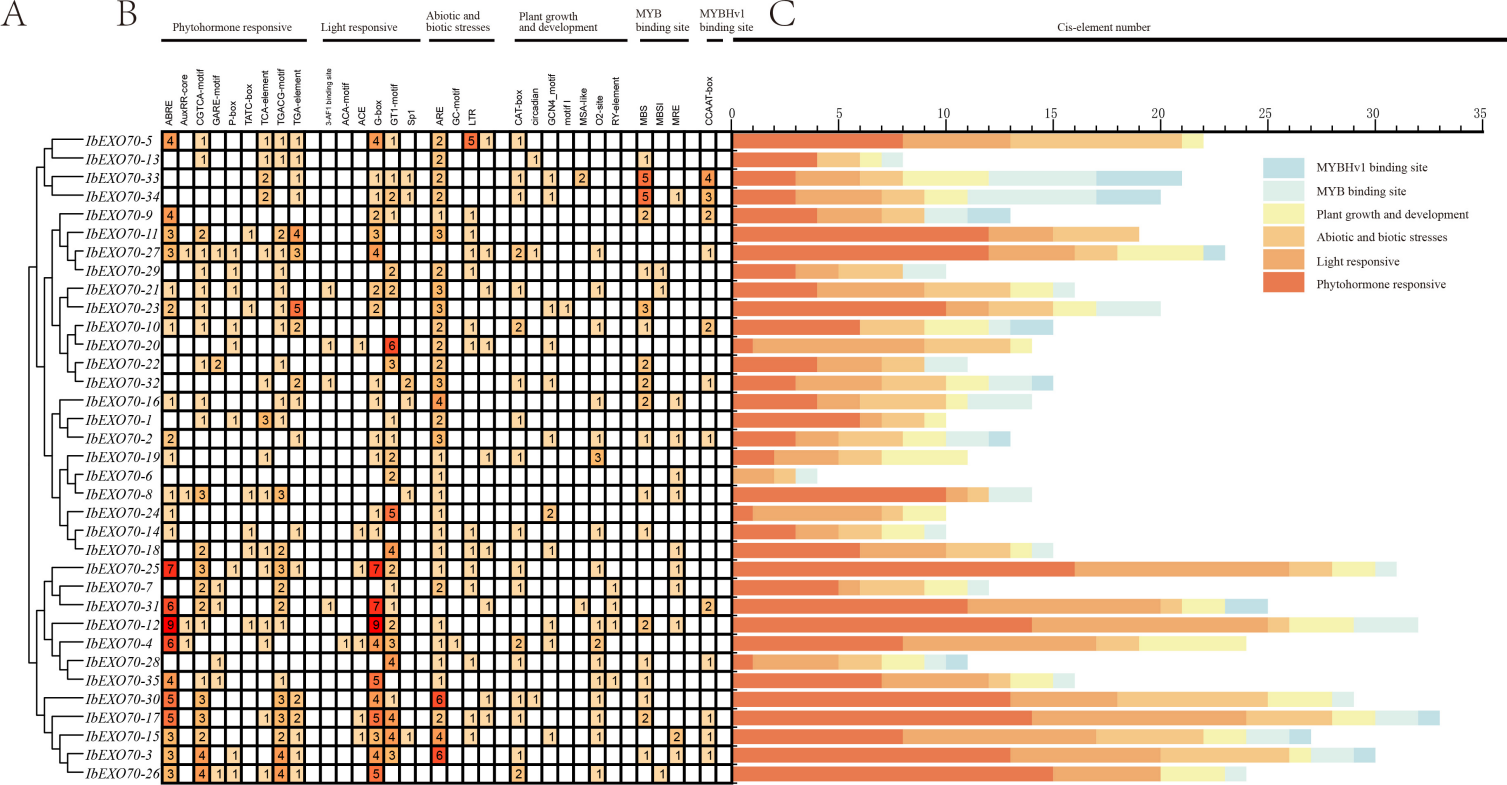
Analysis of *cis*-acting elements in the promoters of *I*. *batatas EXO70* genes. **(A)** The phylogenetic tree of *EXO70* members in *I*. *batatas*. This tree shows the evolutionary relationships of the *IbEXO70* genes, providing a basis for understanding their functional divergence. **(B)** Distribution of *cis*-acting elements in *IbEXO70* promoters highlighting the presence and location of various cis-acting elements within the promoter regions of *IbEXO70* genes, which may be involved in regulating gene expression. **(C)** Number of cis-acting elements contained within each *IbEXO70* gene. Bar graph shows the number of different cis-acting elements in the promoter regions of each *IbEXO70* gene, with color blocks representing different classifications of these elements.

To investigate the potential functions of the *IbEXO70* gene and its transcriptional regulatory mechanisms, a 2kb promoter region upstream of each *IbEXO70* gene was extracted. The PlantCARE online tool was then employed to analyze potential cis-regulatory elements within this region (**Figure 4**). A total of 30 *cis*-regulatory elements were identified in the promoter regions of 35 *IbEXO70* genes and classified into five major categories: hormone responsive, light responsive elements, plant growth and development related elements, abiotic and biotic responsive elements, and transcription factor binding sites (**Figure 4B**). Hormone responsive elements were the most abundant category, comprising nine types: ABRE, AuxRR-core, TGA-element, CGTCA-motif, TGACG-motif, GARE-motif, P-box, TATC-box, and TCA-element, which were associated with responses to abscisic acid, auxin, methyl Jasmonate (MeJA), gibberellin, salicylic acid. Except for the *IbEXO70-6*, all *IbEXO70* genes contained at least one plant hormone responsive element in their promoter regions. Among these, ABRE elements involved in abscisic acid signaling were the most prevalent, suggesting a potential role of *IbEXO70* genes in abscisic acid mediated regulatory pathways.

Additionally, multiple light responsive elements were detected, including the 3-AF1 binding site, ACE, G-box, GT1-motif, MRE, and Sp1, suggesting possible involvement of *IbEXO70* genes in light regulated biological processes. Seven *cis*-elements related to plant growth and development were also identified, including CAT-box, circadian, GCN4_motif, motif I, MSA-like, O2-site, and RY-element, which are implicated in meristem expression, circadian rhythm, endosperm expression, root specific regulation, cell cycle control, zein metabolism, and seed specific regulation.

Promoter regions further contained elements associated with stress responses, such as ARE, GC-motif, LTR, and TC-rich repeats, which are involved in anaerobic induction, hypoxia, low temperature stress response, and defense related mechanisms. The presence of these elements suggests that *IbEXO70* genes may participate in abiotic and biotic stress responses. Furthermore, transcription factor binding sites including MBS, MBSI, MRE, and CCAAT-box were identified, which are known to be involved in drought responsiveness and light signaling. Collectively, these results indicate that *IbEXO70* genes are likely regulated by diverse environmental and developmental cues and may participate in a broad range of biological processes.

### 3.5 Collinearity analysis of *EXO70* genes in *I. batatas*

**Figure 5:**
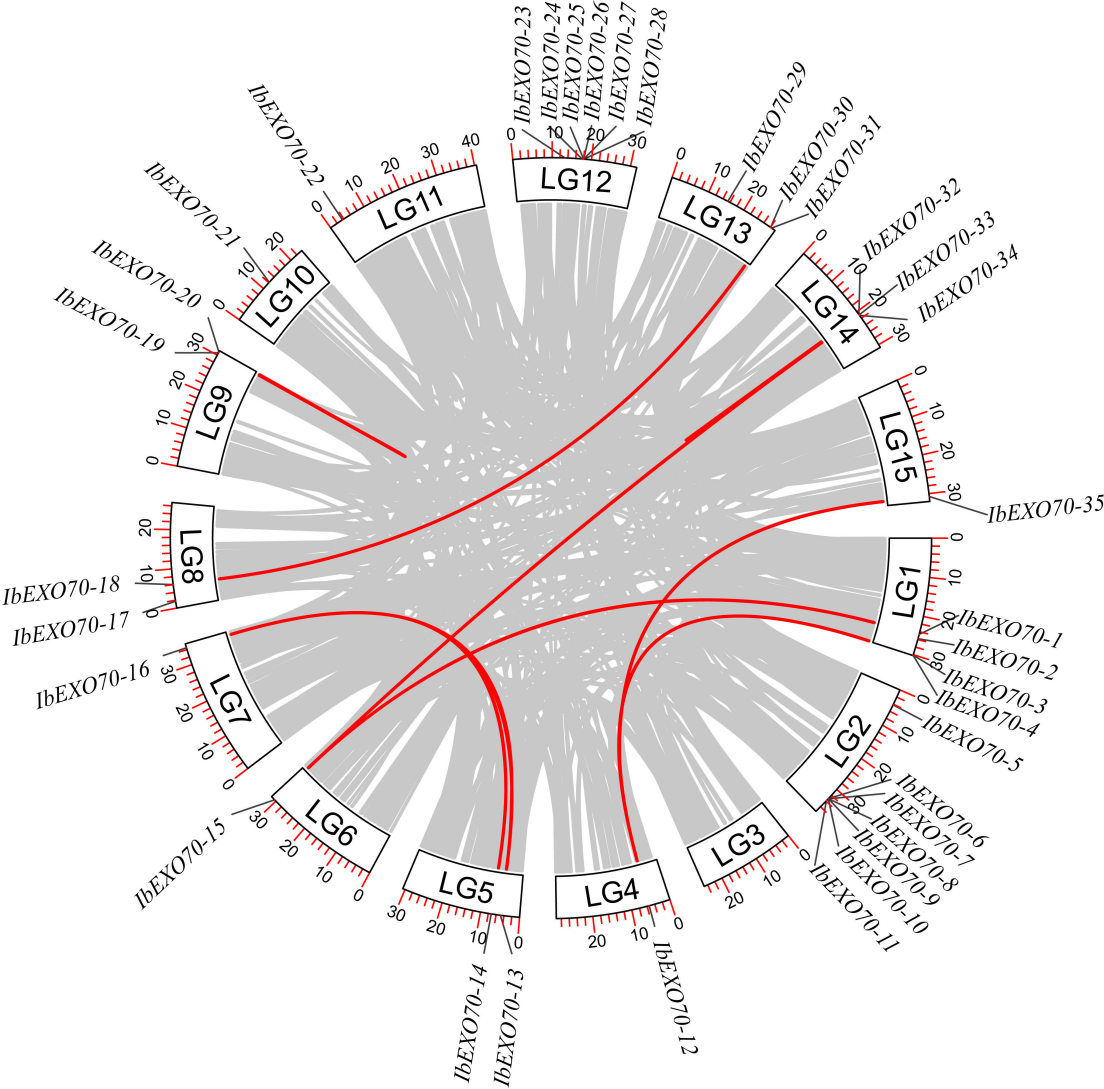
Genome-wide collinearity analysis of *EXO70s* in the *I. batatas* genome. The gray lines represent all the collinear segments in the *I. batatas* genome, and the red lines represent the duplicated *IbEXO70* gene pairs. The white rectangles indicate the chromosomes.

A collinearity analysis of the *EXO70* gene family in *Ipomoea batatas* was performed to investigate their evolutionary relationships and expansion patterns. Our analysis identified both tandem and segmental duplication events. Specifically, two tandem duplication pairs were detected, *IbEXO70-19*/*IbEXO70–20* and *IbEXO70-32*/*IbEXO70-33*. Tandem duplications typically result in the adjacent replication of genes on the same chromosome, which can lead to increased gene dosage or functional divergence. While these tandem duplications contribute to the expansion of the *EXO70* gene family, their role is relatively minor compared to segmental duplications. A total of eight segmental duplication pairs were identified, including *IbEXO70-31*/*IbEXO70-18*, *IbEXO70-13*/*IbEXO70-16*, *IbEXO70-1*/*IbEXO70-15*, *IbEXO70-33*/*IbEXO70-15*, *IbEXO70-34*/*IbEXO70-15*, *IbEXO70-14*/*IbEXO70-16*, *IbEXO70-3*/*IbEXO70-12*, and *IbEXO70-35*/*IbEXO70-12* (**Figure 5**). Segmental duplications typically arise from large scale chromosomal rearrangements and play a more dominant role in the expansion and diversification of gene families. These results underscore that segmental duplications have been the primary mechanism driving the evolution of the *EXO70* gene family in I. batatas, while tandem duplications have contributed to a lesser extent. The collinearity analysis was further visualized in a circular plot, which highlighted the duplications and gene locations across the chromosomes, providing a clear view of the evolutionary dynamics of gene family. These findings contribute valuable insights into the expansion mechanisms of the *EXO70* gene family and its functional diversification in *I. batatas*.

**Figure 6:**
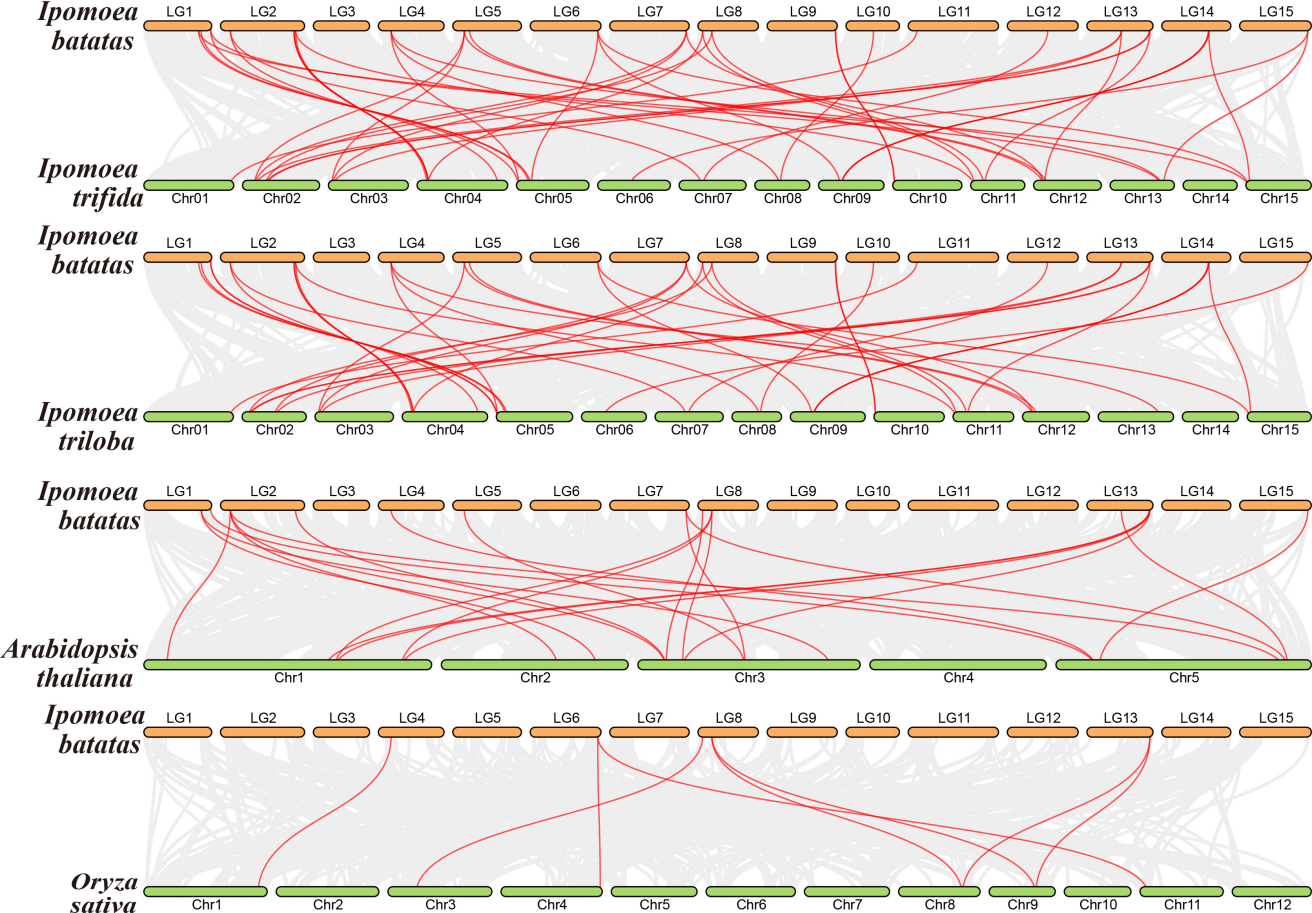
Multi species collinearity analysis of EXO70 genes across different species. This figure illustrates the comparison of the collinearity of *EXO70* genes among *I. batatas*, *I. trifida*, *I. triloba*, *A. thaliana*, and *O. sativa*. The red lines represent homologous gene pairs, demonstrating the evolutionary conservation of the *EXO70* family across these species and highlighting shared genomic regions of duplication.

To further elucidate the evolutionary history and gene family expansion of *EXO70* genes in sweet potato, a comparative synteny analysis was performed across multiple species, including *I. batatas*, *I. trifida*, *I. triloba*, *A. thaliana*, and *O. sativa* (**Figure 6**). Overall, the analysis revealed extensive genomic conservation within the Ipomoea genus. Specifically, we identified 46 orthologous *EXO70* gene pairs were identified between *I. batatas* and *I. trifida*, and 40 orthologous pairs between *I. batatas* and *I. triloba*. This high degree of synteny reflects the shared evolutionary ancestry and conserved genomic architecture among these closely related species. Notably, several of these orthologous pairs corresponded to duplicated *IbEXO70* gene pairs (**Figure 6**) suggesting that segmental duplications have contributed to the expansion of this gene family in sweet potato. In contrast, comparisons with more distantly related species showed a reduced number of homologous *EXO70* pairs. Between *I. batatas* and the dicotyledonous species *A. thaliana* we identified 22 homologous gene pairs, while only eight homologous gene pairs were identified between *I. batatas* and the monocotyledonous species *O. sativa*. These results suggest that the *EXO70* gene family sweet potato shares higher evolutionary conservation with dicotyledonous species than with monocotyledonous plants. Our analyses suggest that the expansion and diversification of *EXO70* genes in sweet potato likely occurred after its divergence from monocots but before its speciation within the Ipomoea genus. The retained syntenic blocks and duplicated gene pairs highlight the role of whole genome or segmental duplication events in shaping the *EXO70* gene repertoire in sweet potato, potentially contributing to its adaptive evolution and functional specialization.

### 3.6 Tissue specific expression dynamics of *IbEXO70* family genes

**Figure 7:**
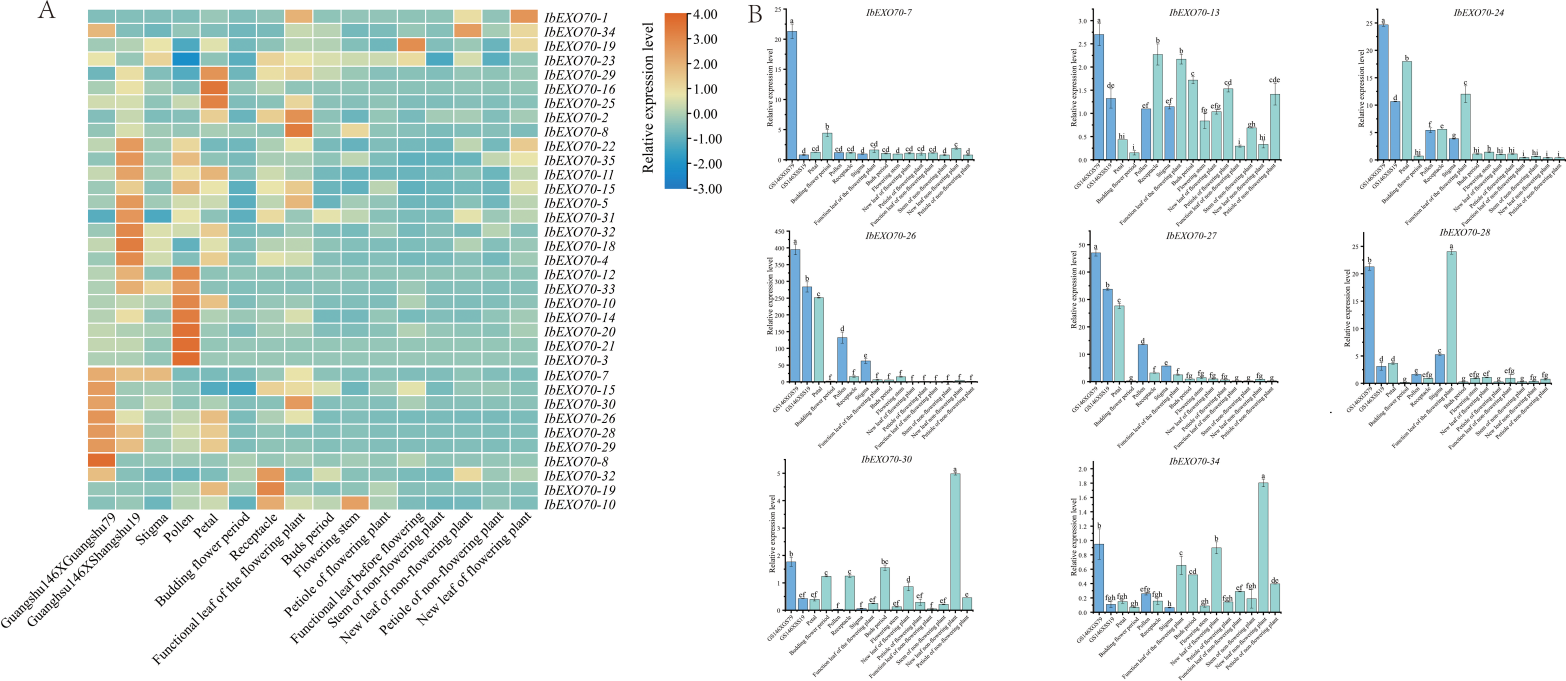
Expression analysis of *IbEXO70* gene in different tissues of *I. batatas.***(A)** Heatmap representing the expression of 35 *IbEXO70* genes across different tissues of *I*. *batatas*. **(B)** Bar graphs representing the expression levels of the eight candidate *IbEXO70* genes involved in ICI regulation. The horizontal axis represents different tissue samples of GS 146. Data was log_2_ transformed, with red blocks indicating higher relative expression levels and blue blocks indicating lower relative expression levels. The lowercase letters on the graphs represent the significance levels of gene expression among different tissues.

To investigate the potential biological roles of *IbEXO70* genes, the expression patterns of all 35 *IbEXO70* family members were examined in various tissues of *I. batatas* using quantitative real-time PCR (qRT-PCR) (**Figure 7A**). Overall, most *IbEXO70* genes exhibited relatively low expression levels budding flower period, buds period, petioles of flowering plants, stems of non-flowering plants, and petioles of non-flowering plants. In contrast, elevated expression of at least one *IbEXO70* gene was detected in each of the remaining tissues examined. Notably, peak expression was observed for 7 *IbEXO70* genes in pollen, 8 genes in stigmas subjected to compatible pollination (GS 146 × GS 79), and 9 genes in stigmas subjected to incompatible pollination (GS 146 × SS 19). These tissue and pollination specific expression patterns suggest that *IbEXO70* genes are actively involved in reproductive processes, particularly in pollen function and stigma responses during pollination, and may play important roles in regulating pollen-stigma interactions in sweet potato.

To further investigate the involvement of *IbEXO70* genes in the regulation of sweet potato ICI regulation, we focused on genes that were significantly upregulated in compatibly pollinated stigmas compared with incompatible or unpollinated stigmas. Based on qRT-PCR results, eight *IbEXO70* genes: *IbEXO70-7*, *IbEXO70-13*, *IbEXO70-24*, *IbEXO70-26*, *IbEXO70-27*, *IbEXO70-28*, *IbEXO70-30*, and *IbEXO70–34* were identified as candidate genes potentially involved in ICI regulation (**Figure 7B**). Among these candidate genes, *IbEXO70-13*, *IbEXO70-30*, and *IbEXO70–34* exhibited relatively low expression levels and were ubiquitously expressed across all 16 tissues examined, suggesting more generalized cellular functions. In contrast, *IbEXO70–26* and *IbEXO70–27* showed the highest expression levels and displayed pronounced tissue specificity, with predominant expression in post pollination stigmas, non-pollination stigmas, pollen, and receptacle tissues, but minimal expression in other examined tissues. This distinct expression pattern indicates that *IbEXO70–26* and *IbEXO70–27* are likely to play specialized roles in reproductive development and pollen-stigma interactions, making them strong candidates for involvement in sweet potato ICI.

**Figure 8:**
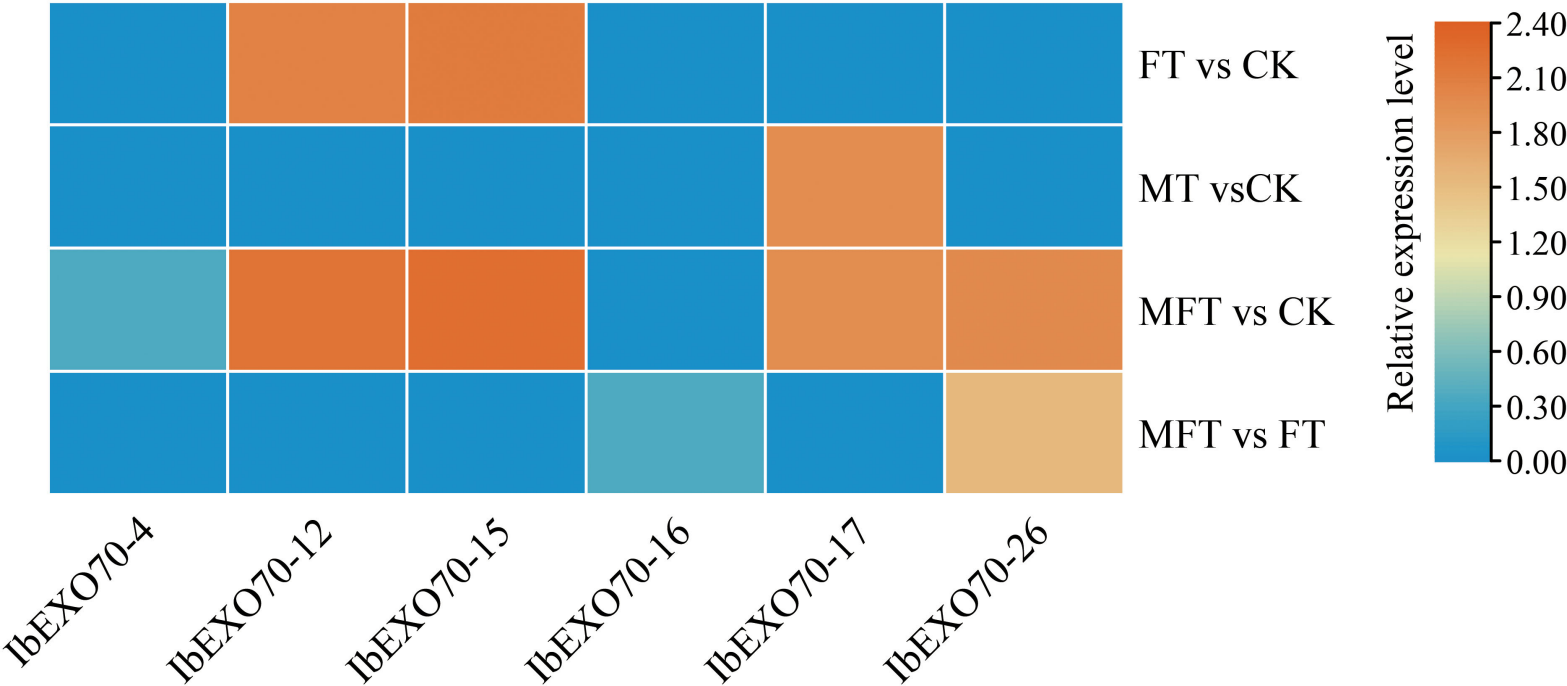
Transcriptome based heatmap for the differential expression of *EXO70* genes in different samples. This heatmap shows the differential expression of *EXO70* genes in various sample groups. The vertical axis represents different sample groups, with red blocks indicating higher expression levels and blue blocks indicating lower expression levels, highlighting patterns of gene regulation.

In a previous transcriptomic analysis of sweet potato intraspecific cross-incompatibility (ICI), six *IbEXO70* genes were identified using four types of stigma samples: CK (unpollinated stigma), MT (stigma treated by a cross-incompatibility overcoming reagent), FT (incompatible pollinated stigma) and MFT (compatible pollinated stigma) (Yang et al., 2022). These genes included *IbEXO70-4*, *IbEXO70-12, IbEXO70-15, IbEXO70-16*, *IbEXO70-17, IbEXO70-26* (**Figure 8**). Among these genes, all exhibited differential expression in MFT compared with CK, except *IbEXO70-16*. However, *IbEXO70–26* was the only gene that showed significant differential expression in MFT compared with FT, whereas no significant expression differences were detected in FT vs CK and MT vs CK. This unique expression pattern suggests that *IbEXO70–26* is specifically associated with compatible pollination rather than general stigma activation or stress responses. Therefore, these transcriptomic data further support *IbEXO70–26* as a key candidate gene involved in the regulation of sweet potato intraspecific cross-incompatibility.

### 3.7 The subcellular localization of *IbEXO70-26*

**Figure 9:**
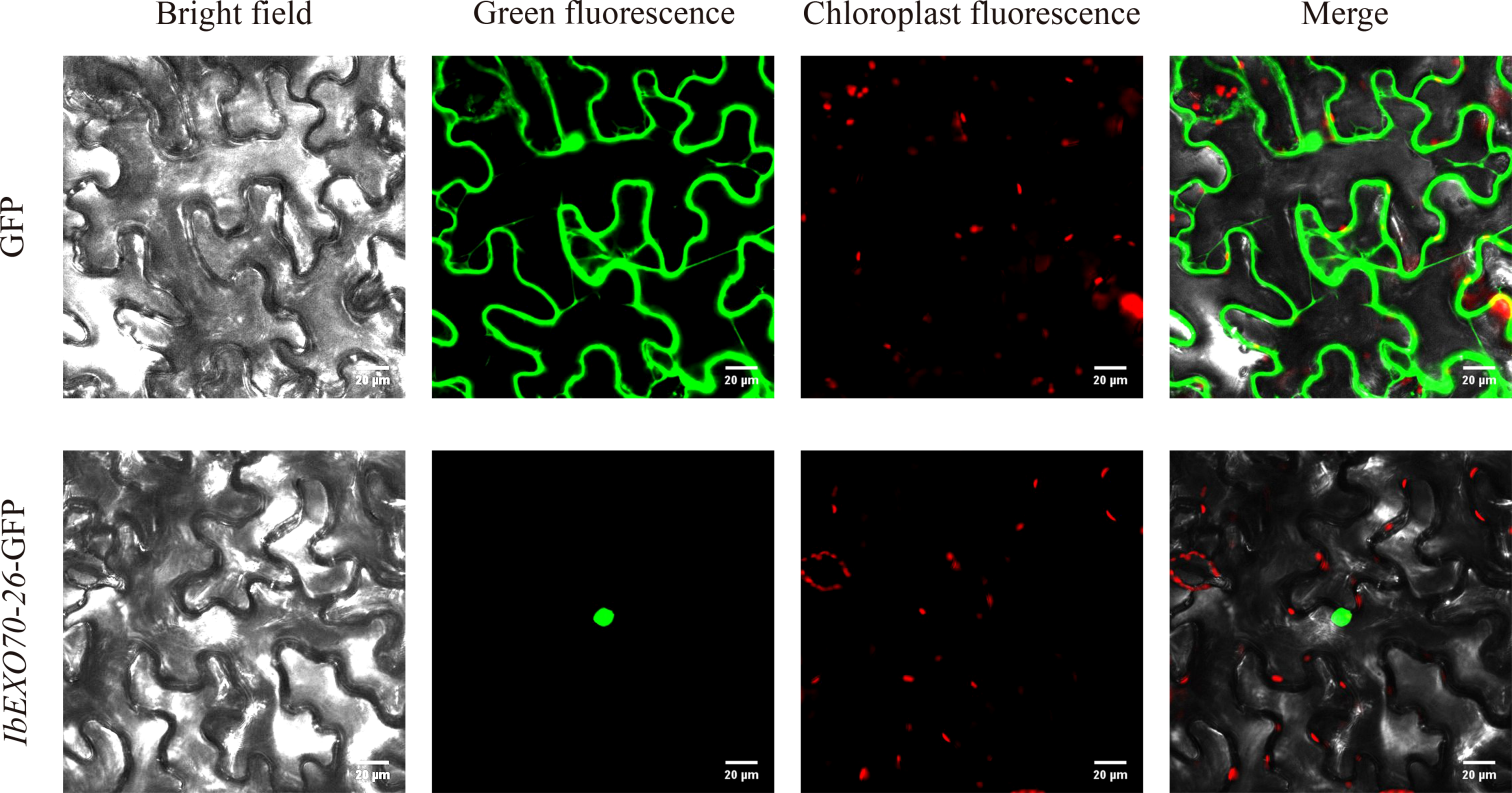
Subcellular localization of *IbEXO70-26*. The subcellular localization of *IbEXO70–26* was determined using GFP fusion protein technology. The free GFP control showed fluorescence signals in the cytoplasm, cell membrane, and nucleus. Red color indicates autofluorescence from chloroplasts. Scale bar: 20 μm, providing the size reference for observed structures and fluorescence patterns.

To investigate the potential function of *IbEXO70-26*, its full length coding sequence was cloned and fused to green fluorescent protein (GFP) and subjected to subcellular localization analysis. The *bEXO70-26*–GFP fusion construct was transiently expressed in *Nicotiana benthamiana* leaves, with free GFP used as a control. As expected, the free GFP control showed fluorescence signals in the cytoplasm, cell membrane, and nucleus. In contrast, the fluorescence signals from the IbEXO70-26-GFP fusion protein were exclusively detected in the nucleus (**Figure 9**), indicating that *IbEXO70–26* is predominantly localized to the nucleus.

## 4 Discussion and conclusion

In this study, the evolutionary relationships of the *EXO70* gene family in sweet potato were investigated based on gene number, chromosomal distribution, structural features and classifications. A total of 94 *EXO70* genes were identified across three *Ipomoea* species: including 35 genes in hexaploid *Ipomoea batatas*, 30 and 29 genes in the diploid species *Ipomoea trifida*, and *Ipomoea triloba*, respectively. It is different from cotton (Zhu et al., 2021) and wheat (Zhao et al., 2018), in which the number of *EXO70* genes increases via genome polyploidization and indicates a high degree of conservation of the *EXO70* family genes between hexaploid and diploid *Ipomoea* species. It may be attributed to the high homology among the six sets of genomes in hexaploid *I. batatas* and the absence of gene replication during polyploidization, while the chromosome number increased (Pikaard, 2001; Adams and Wendel, 2005; Buggs et al., 2009; Ma et al., 2024). Nevertheless, there is a distinct difference in chromosomes distribution of *EXO70* genes. The *EXO70* genes in *I. trifida* and *I. triloba* were primarily distributed on chromosomes 2, 4, 5, and 7, but the *EXO70* genes in *I. batatas* were mainly concentrated on chromosomes 1, 2, 12, 13, and 14. It may be due to chromosomal rearrangements, segmental duplications and gene translocation during polyploid evolution (Harewood and Fraser, 2014; Augustijnen et al., 2024) and may result in different biological functions. These findings are consistent with the hypothesis that sweet potato is an auto-hexaploid or hexaploid species (Yang et al., 2017).

Phylogenetic analysis classified *EXO70* genes from *Ipomoea* species into three major clades (EXO70.1-EXO70.3) and nine subgroups (EXO70A-EXO70I), which is consistent with previous studies in other plant species (Zhao et al., 2018; Liu et al., 2023). The distribution of genes among the EXO70A-H subgroups and structural construction was highly similar between cultivated sweet potato and its wild relatives, further demonstrating the conservation of *EXO70* genes in *Ipomoea* species. However, compared to *A. thaliana*, cotton (Zhu et al., 2021), cucumber (Liu et al., 2023), EXO70A subgroup in *Ipomoea* species was effectively amplified, especially for IbEXO70A subgroup possessing 10 genes. Similar to the other plant species, members of EXO70A subgroup consistently contained higher number of exons. Collinearity analysis revealed that both tandem and segmental duplications contributed to the evolution of the *IbEXO70* gene family and segmental duplication was the predominant mechanism. Interestingly, there were one in two tandem duplication pairs, three in eight segmental duplication pairs from IbEXO70A subgroup. EXO70A is engaged in the canonical exocyst function in polarized exocytosis which is important for polar growth and cell wall biogenesis (Marković et al., 2021). It implies *IbEXO70A* genes may play an important role in sweet potato development and growth. Gene promoters, which are DNA sequences located upstream of the coding region, regulates gene expression through *cis*-acting elements and plays a key role in plant growth and stress responses (Hernandez-Garcia and Finer, 2014). Promoter analysis further provided insights into the regulatory potential of *IbEXO70* genes. Numerous *cis*-acting elements related to plant hormones signaling were identified, particularly ABRE elements associated with ABA signaling and abiotic stress responses (Narusaka et al., 2003; Du et al., 2023). In addition, light responsive and stress responsive elements were also identified in the *IbEXO70* promoters. These findings suggest that *IbEXO70* genes are regulated by diverse environmental and developmental cues and may participate in multiple biological processes, including stress adaptation and reproductive development.

Expression profiling across different tissues revealed that several *IbEXO70* genes were preferentially expressed in reproductive organs, particularly pollen and stigmas. Importantly, nine *IbEXO70* genes exhibited significant differential expression between compatible and incompatible pollinated stigmas. Among these, *IbEXO70–26* exhibited specific and elevated expression in compatible stigmas, with low expression levels in other tissues. Transcriptomic differential analysis further confirmed that *IbEXO70–26* was the only *EXO70* gene showing significant expression differences between the MFT and FT comparison groups. This unique expression pattern strongly suggests a specific role for *IbEXO70–26* in regulating intraspecific cross-incompatibility (ICI) in sweet potato. In *Brassicaceae*, *EXO70A1* has been identified as a compatibility factor, with loss of function mutations disrupting pollen hydration and inhibiting pollen tube elongation (Samuel et al., 2009; Safavian et al., 2014). However, *IbEXO70–26* belongs to the EXO70H subgroup and exhibits significant expression differences in compatible stigmas compared to *IbEXO70–7* and *IbEXO70-34*, which belong to the EXO70A subgroup. Previous functional studies of *EXO70* genes in *A. thaliana* showed that, *EXO70H1* is involved in response to pathogens (Pecenkova et al., 2011). *EXO70H3*, *EXO70H5*, *EXO70H6* are highly expressed in pollens (Li et al., 2010; Zarsky et al., 2020), while *EXO70H4* has a specific role in callose synthase secretion (Kulich et al., 2015; Kulich et al., 2018). Callose deposition in stigma papilla cells has been shown to be a critical factor inhibiting pollen germination and triggering ICI in sweet potato (Yiling et al., 2018). Additionally, the *IbEXO70–26* gene contains MYB transcription factor binding sites, and MYB transcription factors have been reported to be essential for normal pollen germination and seed set (Leydon et al., 2013; Meng et al., 2018; Wang et al., 2023; Wang et al., 2024), whereas *IbEXO70A* genes are primarily enriched in light responsive elements. These observations collectively suggest that *IbEXO70-26*, as a member of the EXO70H subgroup, may function as an alternative compatibility response factor by modulating pollen-stigma recognition signals and influencing pollen tube growth during compatible pollination. Previous studies have largely indicated that the EXO70 protein primarily localizes to the cytoplasm and cell membrane, where it functions in exocytosis, vesicular transport, and polar growth. These processes are closely associated with pollen hydration and pollen tube development within the self-incompatibility response (Samuel et al., 2009; Sekereš et al., 2017; Synek et al., 2021). However, in this study, IbEXO70–26 was specifically localized within the cell nucleus, representing a significant deviation from the classical localization pattern observed in most EXO70 family members. This is consistent with the established localization of EXO70H in tobacco and *A. thaliana* to the cell nucleus (Zarsky et al., 2020). It is speculated that the *IbEXO70–26* gene exerts an atypical secretory function in regulating sweet potato ICI, instead playing a role in signal transduction and transcriptional regulation, thereby influencing the expression of downstream compatibility or incompatibility genes.

In summary, this study provides a comprehensive analysis of the evolutionary characteristics, structural features, expression patterns, and potential functions of the *EXO70* gene family in sweet potato. Our results identify *IbEXO70–26* as a strong candidate gene involved in the regulation ICI in sweet potatoes. Although further functional validation is required, his work establishes a solid foundation for future studies, including genetic transformation and molecular dissection of *IbEXO70-26*, to elucidate its precise role in sweet potato reproductive compatibility.

## Data Availability

The datasets presented in this study can be found in online repositories. The names of the repository/repositories and accession number(s) can be found in the article/**Supplementary Material**.
