# Supplementary material for: Genome-wide identification of the EXO70 genes to elucidate their potential roles for intraspecific cross-incompatibility in sweet potato (Ipomoea batatas L.)
Source: Front Plant Sci. 2026 Feb 26;17:1756265. doi: 10.3389/fpls.2026.1756265 (PMC12979542; doi:10.3389/fpls.2026.1756265)
Supplement: Supplementary file 1 [file Presentation1.zip › Figure.pptx]

## Slide 1
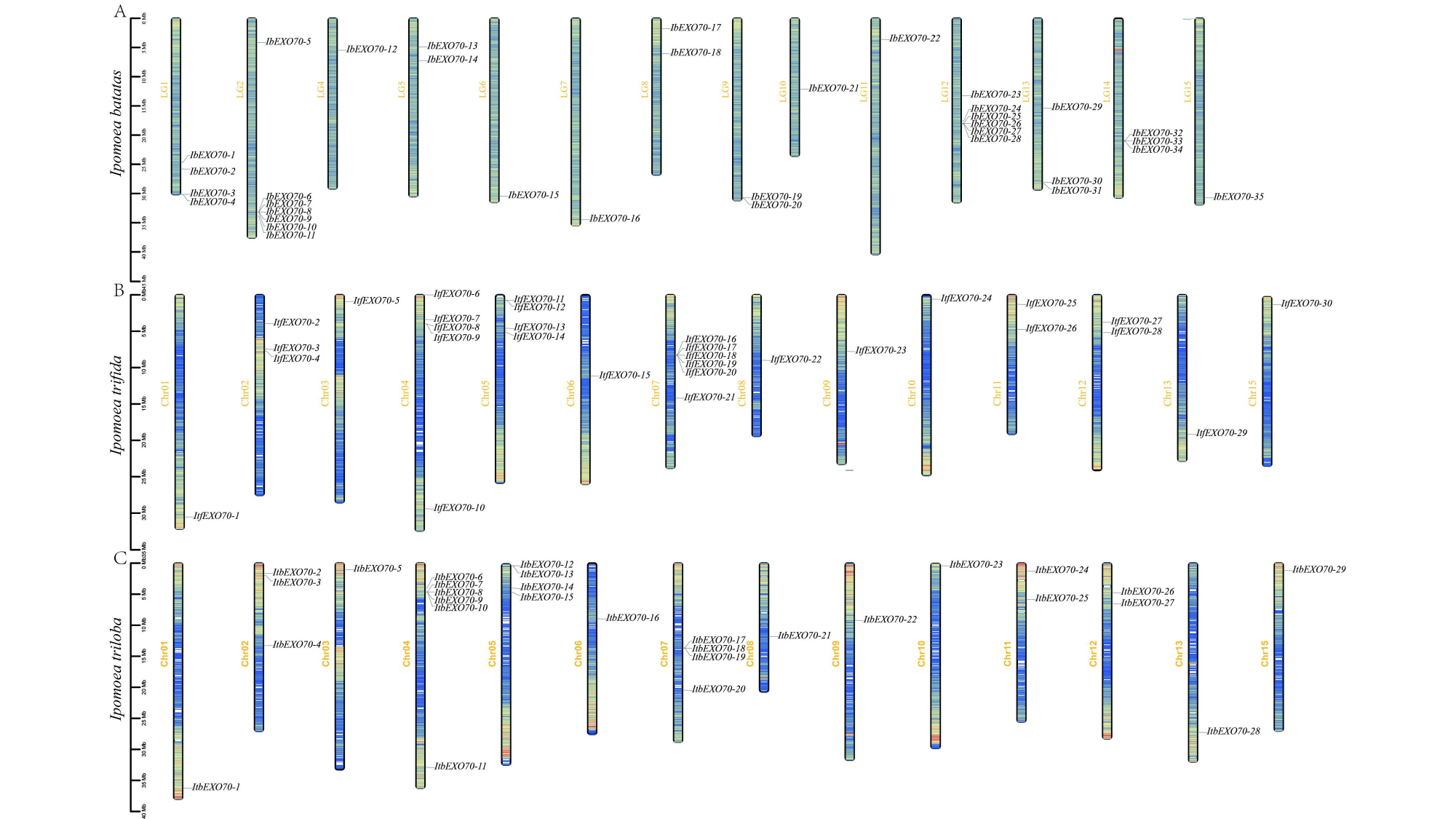

Figure 1. Chromosomal distribution of EXO70 gene family members in three Ipomoea species.

## Slide 2
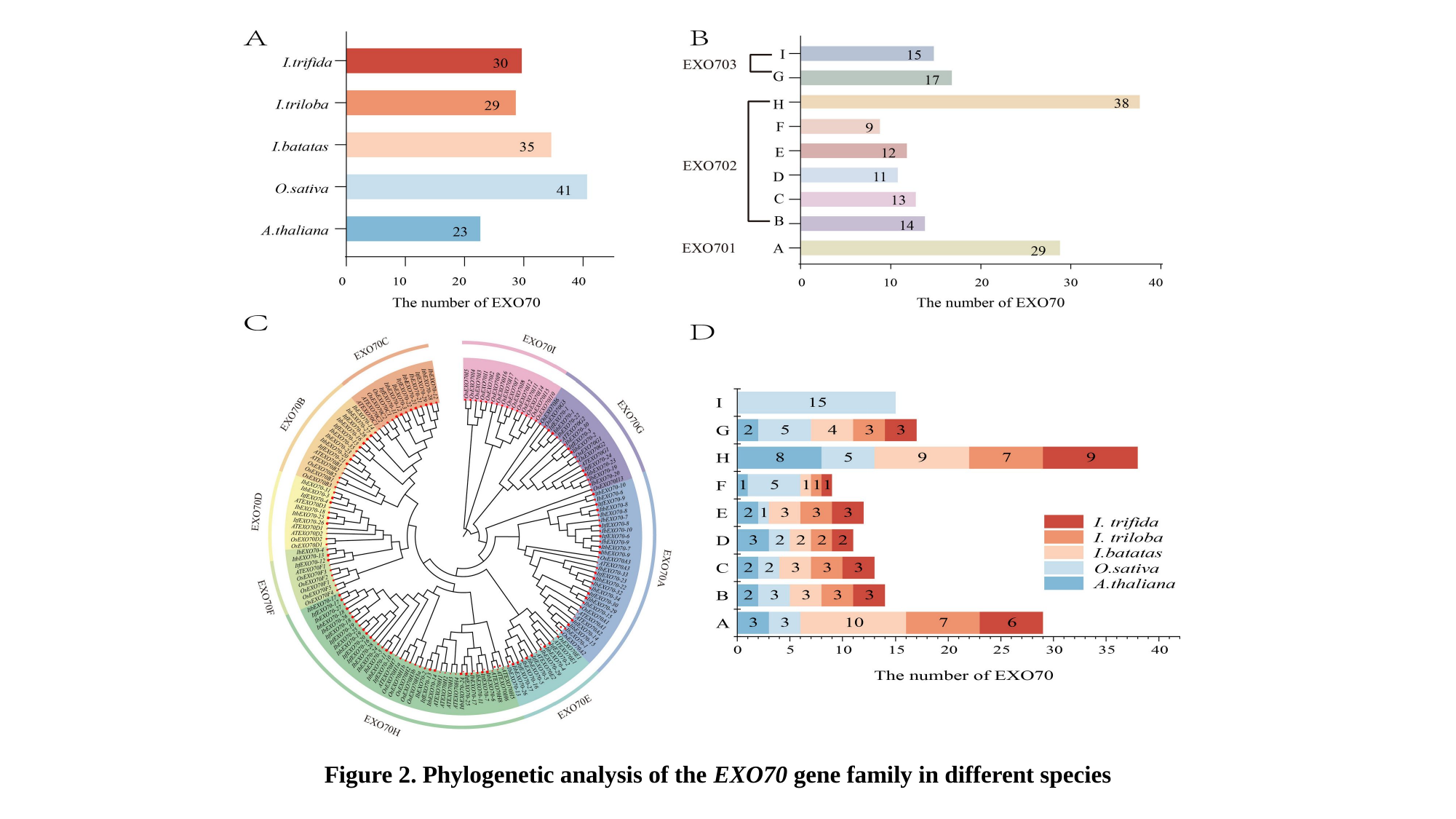

Figure 2. Phylogenetic analysis of the EXO70 gene family in different species

## Slide 3
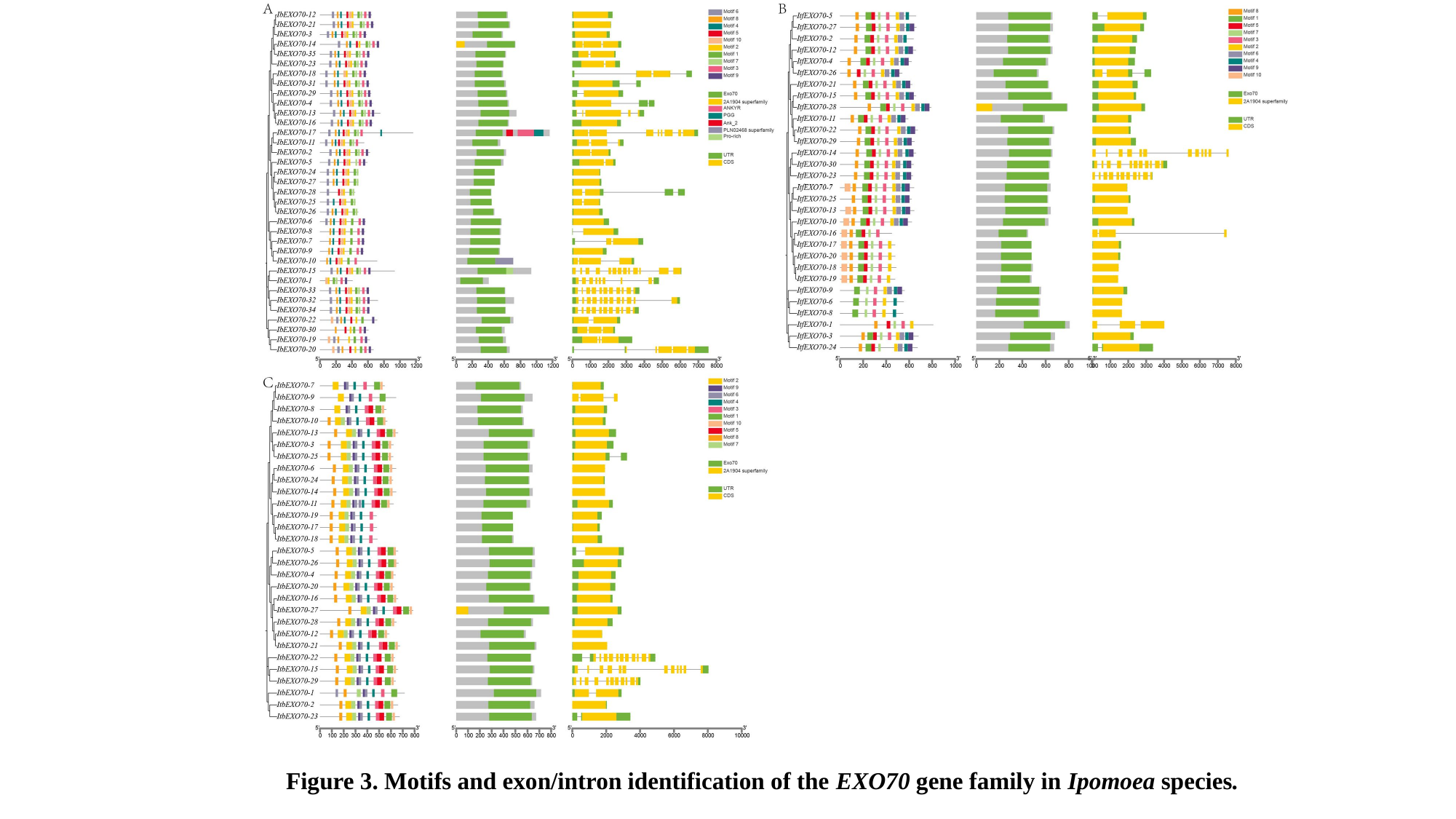

Figure 3. Motifs and exon/intron identification of the EXO70 gene family in Ipomoea species.

## Slide 4
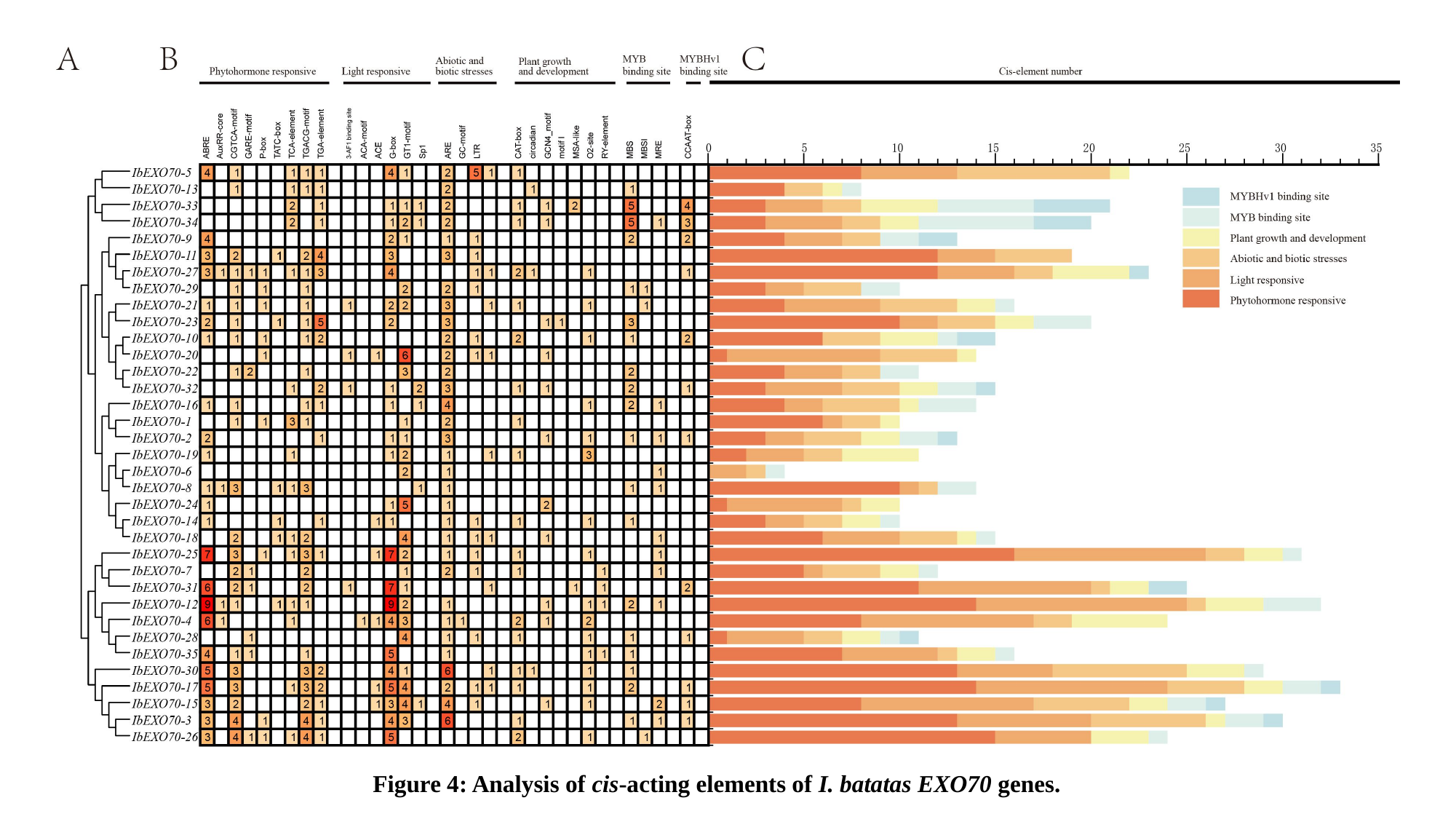

Figure 4: Analysis of cis-acting elements of I. batatas EXO70 genes.

## Slide 5
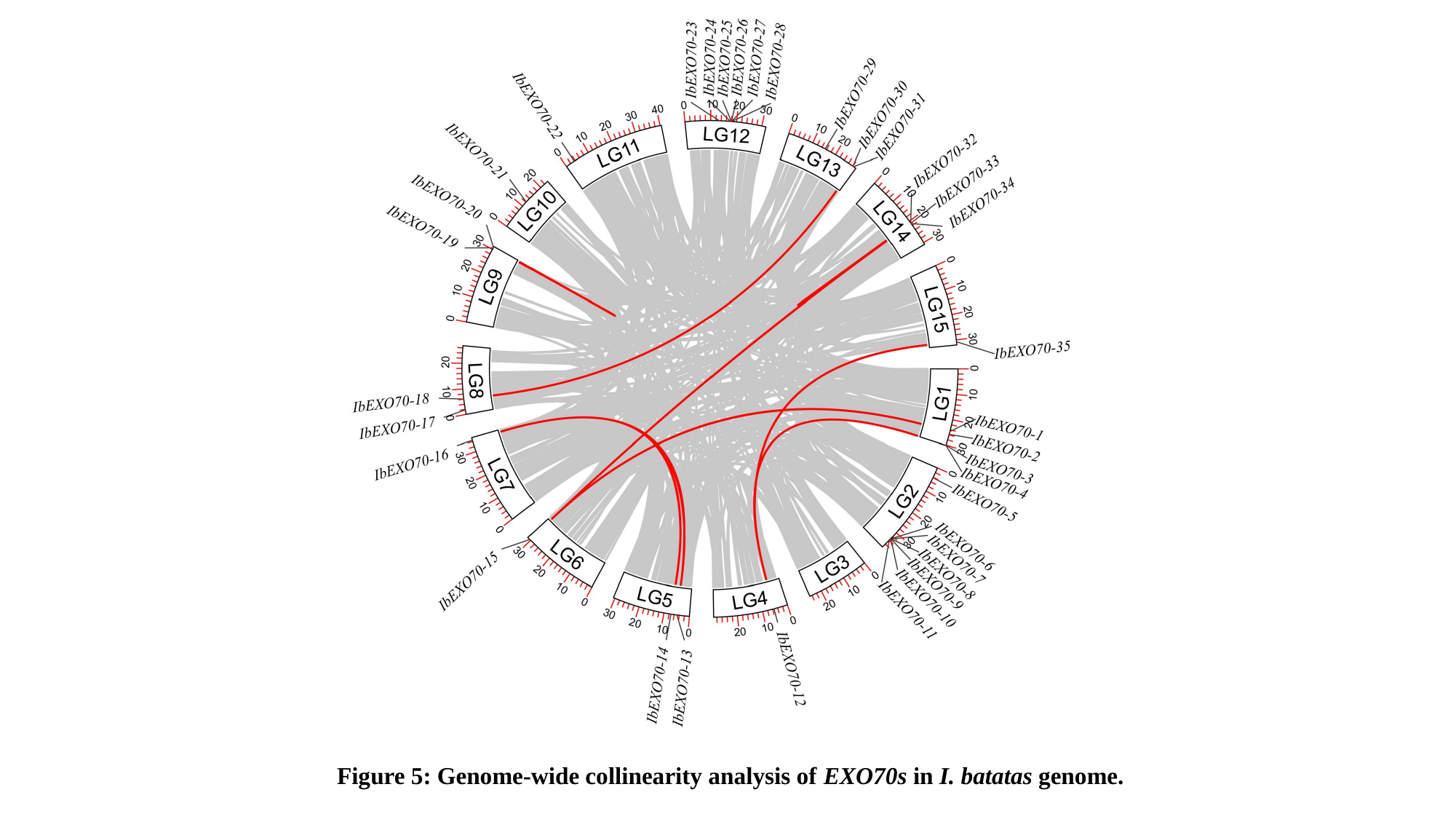

Figure 5: Genome-wide collinearity analysis of EXO70s in I. batatas genome.

## Slide 6
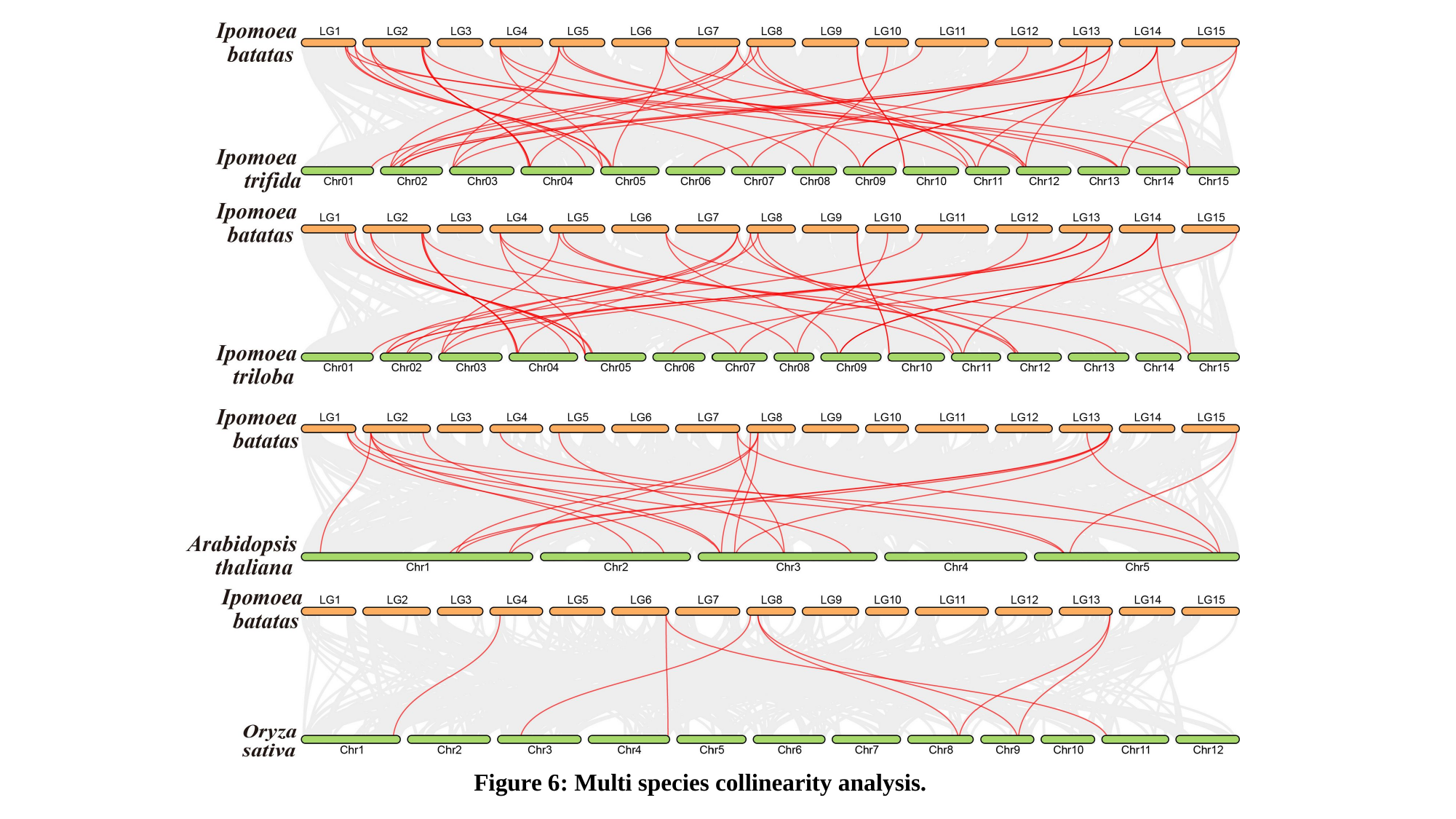

Figure 6: Multi species collinearity analysis.

## Slide 7
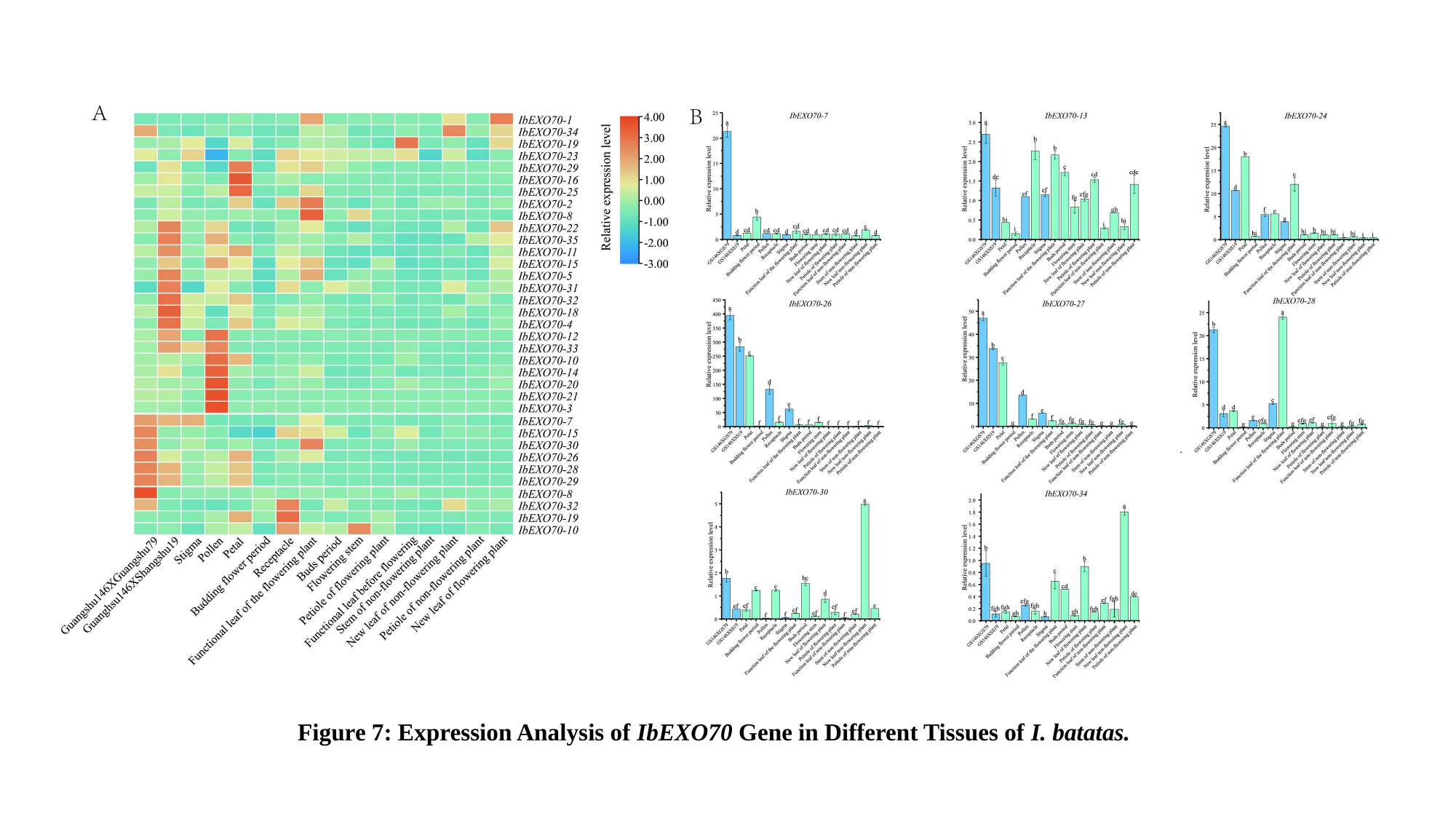

Figure 7: Expression Analysis of IbEXO70 Gene in Different Tissues of I. batatas.

## Slide 8
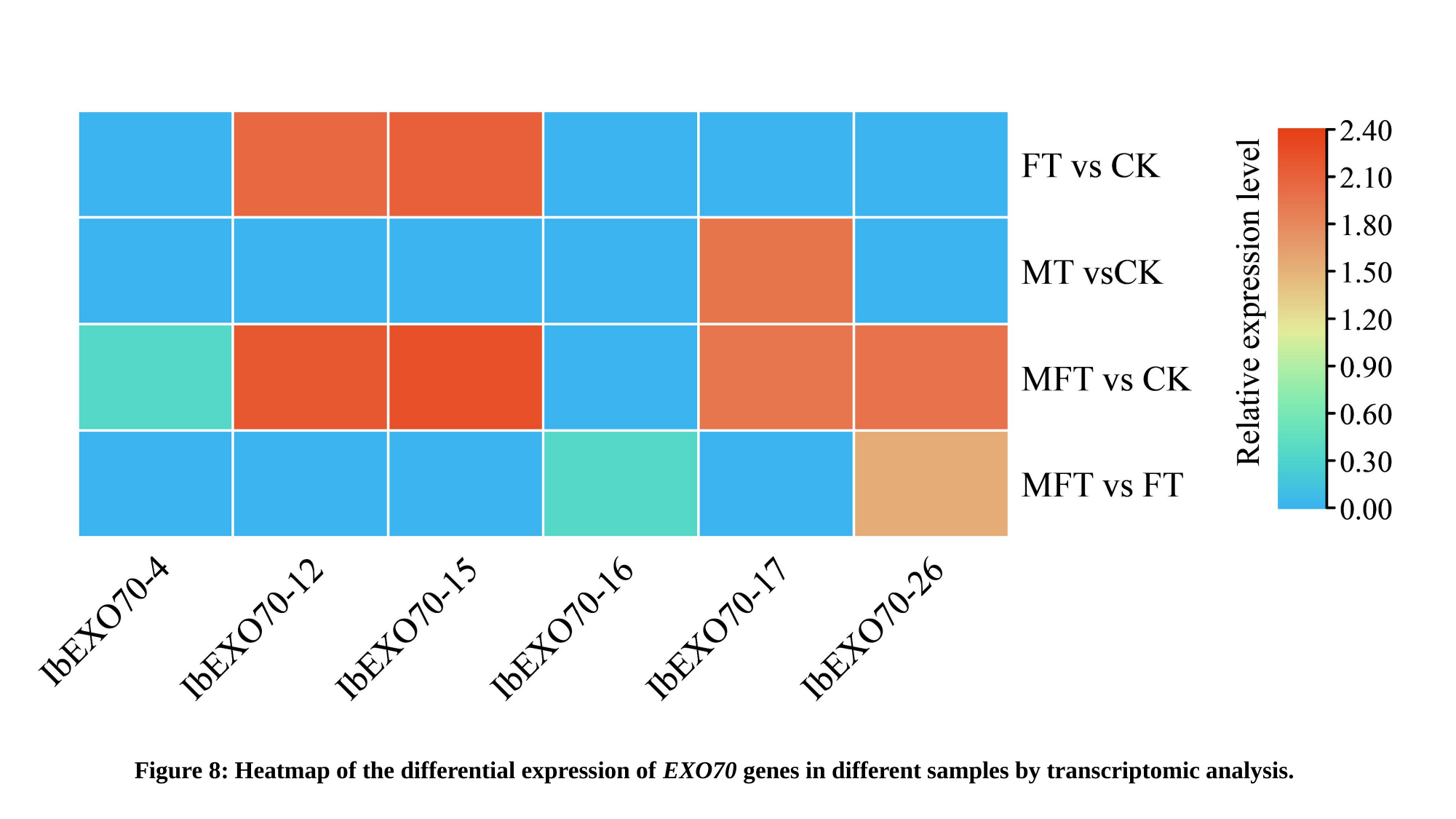

Figure 8: Heatmap of the differential expression of EXO70 genes in different samples by transcriptomic analysis.

## Slide 9
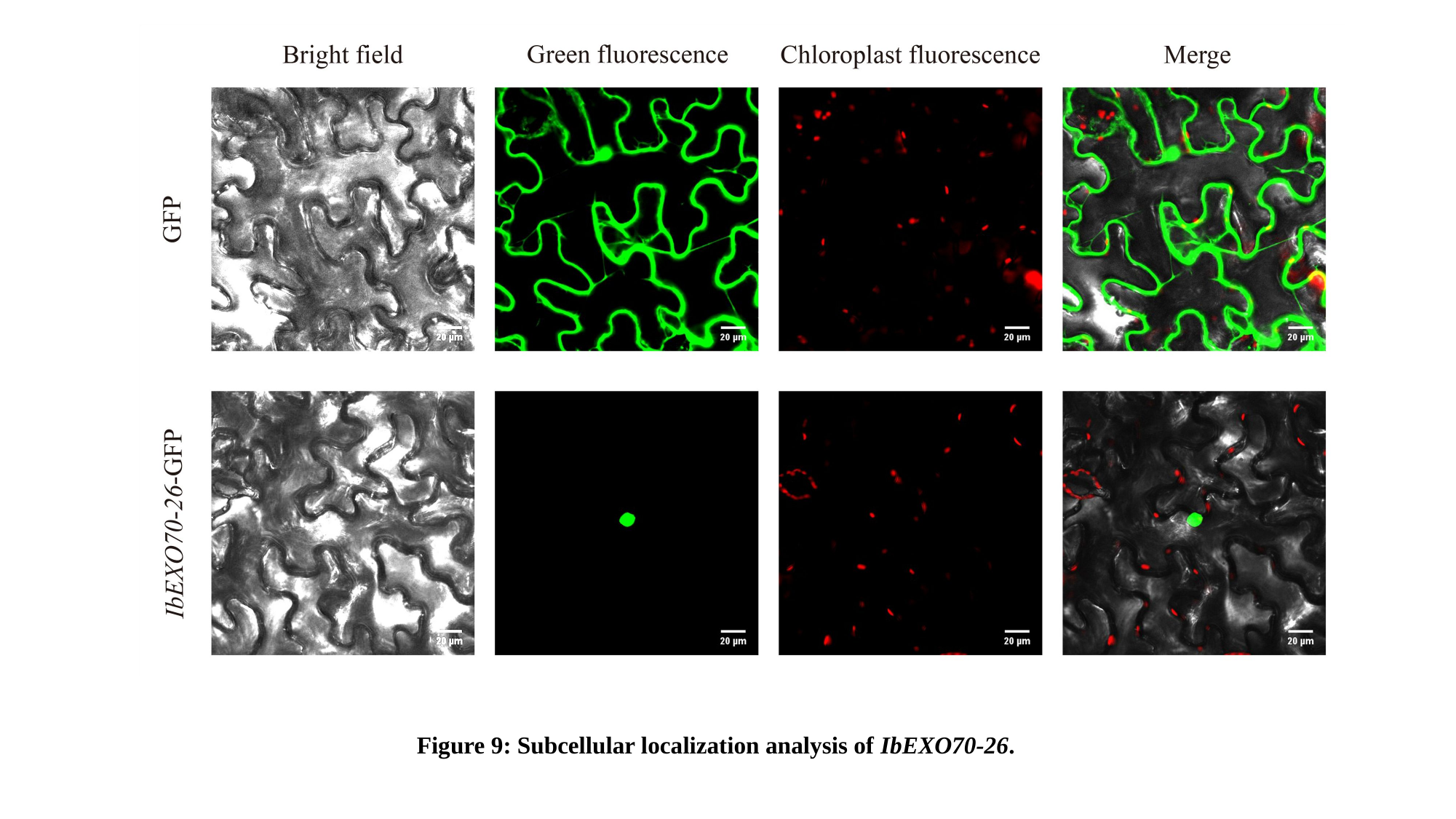

Figure 9: Subcellular localization analysis of IbEXO70-26.
